# EndoTac: An Endoscopic Camera‐Based Tactile Sensor with High Sensitivity for Minimally Invasive Surgery

**DOI:** 10.1002/advs.76921

**Published:** 2026-07-31

**Authors:** Yupeng Wang, Nicolas Raison, Xuyang Zhang, Aijia Yang, Lukas Lindenroth, Shan Luo

**Affiliations:** ^1^ Department of Engineering King's College London London UK; ^2^ School of Biomedical Engineering & Imaging Sciences King's College London London UK; ^3^ King's College Hospital London UK; ^4^ Faculty of Engineering Imperial College London London UK

**Keywords:** deformation sensing, force sensing, minimally invasive surgery, tactile sensing, vascular sensing

## Abstract

Minimally invasive surgery (MIS) removes direct tactile feedback from surgical procedures, making it challenging for surgeons to manipulate delicate anatomy, such as vasculature and other soft tissue. Existing tactile tools developed for MIS in prior research are either too bulky or have limited sensing coverage, restricting their utility in practical surgical workflows. To address these challenges, we introduce EndoTac, a novel miniaturized vision‐based tactile sensor designed to traverse a standard trocar while offering a large sensing area with high sensitivity. EndoTac integrates an endoscopic camera to achieve a compact and MIS‐compatible form factor, and employs a convex mirror to capture the contact information across a wide sensing area, thereby maximizing effective sensing coverage. A soft membrane further enables the sensor to achieve high sensitivity by deforming under gentle interactions with soft and fragile tissue like vascular structures. Experimental results demonstrate that EndoTac achieves a low detection threshold of 20.5 mN, a high pixel‐level sensitivity slope in the low‐force regime, improved force resolution, and reliable estimation of vessel deformation in vascular palpation tests. Together, these results show that EndoTac provides a significant step toward restoring tactile perception in MIS, offering a practical pathway to safer and more precise surgical manipulation.

## Graphical abstract

EndoTac presents a trocar‐compatible endoscopic vision‐based tactile sensor that uses a convex mirror to enlarge side‐facing tactile coverage during minimally invasive vessel palpation. Distorted tactile images are unwarped and processed by a learning model to estimate vascular deformation, enabling sensitive, spatially distributed tactile perception for safer interaction with delicate soft tissue.

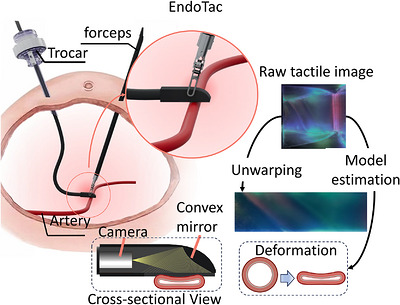

## 1 Introduction

MIS has revolutionized modern surgical practice by reducing patient trauma, reducing hospital stays, and improving recovery times [1, 2]. However, by replacing direct hand‐tissue interaction with long, slender instruments inserted through small ports, MIS inherently removes the surgeon's natural tactile perception [3, 4]. This loss of touch makes it significantly more challenging to judge tissue compliance, detect subtle changes in vessel stiffness, or regulate the delicate forces required when manipulating fragile structures. As a result, surgeons rely on visual cues to infer contact conditions, increasing cognitive load and the risk of inadvertent tissue damage, suboptimal grasping, and surgical complications [5].

In MIS, sensing or estimating soft‐tissue deformation is important because tissue displacement provides indirect information about tool–tissue interaction, applied force, tissue stiffness, and the risk of mechanical damage. Therefore, accurate tracking of tissue deformation has been recognized as an important component of computer‐assisted MIS, particularly for surgical guidance, motion compensation, navigation, and the registration of preoperative information in the deformed intraoperative scene [6, 7, 8]. However, unlike open surgery, MIS limits direct tactile perception, requiring surgeons to infer tissue loading and deformation mainly from visual feedback.

Among different forms of soft tissue interaction, vessel manipulation represents a particularly challenging and clinically important task because vessels are compliant, delicate, and mechanically sensitive to localized compression, traction, and displacement. This is especially relevant during procedures involving vessel dissection, tumor resection, or vascular repair, where surgeons must continuously assess how vessels deform in response to applied instrument forces.

For this reason, vascular deformation estimation requires the recognition of subtle changes in vessel wall displacement, stiffness, and lumen compression, all of which provide important cues for the safe manipulation of fragile vascular structures. In the absence of direct tactile feedback, surgeons must infer tool–vessel interactions indirectly, making it difficult to determine both the magnitude of the applied force and the extent of vessel deformation. This limitation increases the risk of excessive compression, which may cause partial or complete arterial collapse and consequently increase the likelihood of inadvertent vessel injury or intraoperative hemorrhage [9]. Therefore, reliable vascular deformation sensing could provide surgeons with more direct information about the mechanical state of vessels, supporting safer, more precise, and more controlled interaction with delicate vascular structures.

In current MIS workflows, vascular assessment is typically performed using visual inspection or adjunct imaging such as Doppler ultrasound. However, these modalities often lack sensitivity needed to detect small deformations and provide feedback that is difficult to interpret, as imaging data are not naturally aligned with the surgeon's frame of reference [10, 11]. As a result, surgeons must infer mechanical cues indirectly, increasing cognitive burden and reducing precision.

Introducing tactile sensing into the MIS environment offers a direct and intuitive means to estimate applied forces and the resulting vascular deformation, enabling safer and more controlled manipulation of delicate vascular structures [12].

This need is consistent with recent reviews of MIS and robot‐assisted MIS, which identify the loss of tactile and force feedback as a persistent limitation for tissue handling, palpation, surgical training, and safe tool–tissue interaction [13, 14, 15, 16]. More broadly, recent advances in sensor‐integrated medical tools have shown that miniaturized sensors can be incorporated into needles, catheters, endoscopes, and robotic surgical end‐effectors to recover local physical information that is otherwise unavailable during minimally invasive access [17].

Overall, existing MIS tactile systems provide valuable force, stiffness, grasping, thickness, slip, contact state, and haptic feedback information [1, 18, 19, 20, 21, 22, 23, 24]. However, many of these systems remain primarily force or stiffness‐oriented. For vessel palpation, the contact and deformation may extend along the vessel wall rather than being confined to a small contact point. This creates a need for a compact tactile sensor that can observe spatially distributed deformation while remaining compatible with MIS dimensional constraints. This motivates the sensing area/cross‐sectional area ratio (SA/CA) as a useful design metric for miniaturized MIS tactile sensors. For a fixed trocar‐compatible probe cross‐section, a higher SA/CA indicates that a larger usable tactile area can be achieved without increasing the probe diameter.

**FIGURE 1:**
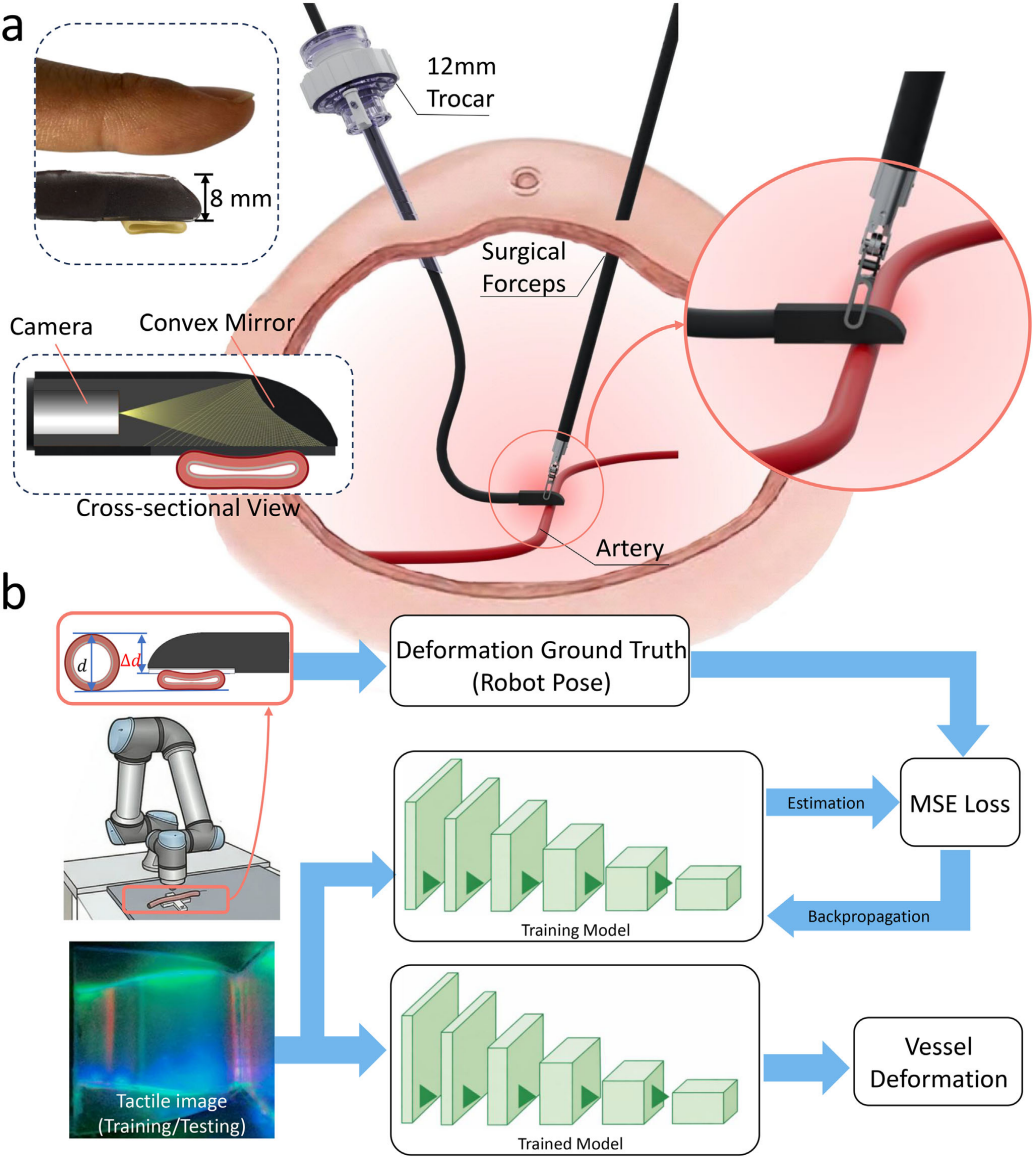
Overview of the EndoTac sensing concept and deformation estimation pipeline. (a) Illustration of the EndoTac probe deployed through a 12 mm trocar and manipulated using laparoscopic forceps. The cross‐sectional view shows the internal optical configuration, where a convex mirror redirects the imaging path from the side‐facing elastomer sensing surface to the endoscopic camera, enabling wide area tactile sensing while maintaining a compact probe geometry with the help of the cylindrical convex mirror. (b) Tactile perception and deformation estimation pipeline. Raw tactile images captured through the convex mirror are used to construct a training dataset together with deformation ground truth obtained from sensor displacement from robot pose and vessel size. The tactile images are then processed by a neural network based model to estimate vessel deformation, defined by the relative change in vessel diameter during palpation.

To address these challenges, in this work, we introduce *EndoTac*, a novel endoscopic camera‐based tactile sensor with high sensitivity and a large observable tactile region within a trocar‐compatible size limit. As shown in Figure 1, EndoTac employs a light‐reflective optical design that overcomes the conventional trade‐off between sensor miniaturization and sensing area by introducing a convex mirror design to enlarge the sensing area. Such design enables a compact, trocar‐compatible form factor while preserving a large and effective sensing region. This extended sensing region is particularly important for detecting contact edges and distributed deformation when the probe interacts with soft vascular tissue. In addition, EndoTac adopts a soft membrane selected to improve the visual response under gentle soft‐tissue interactions. This membrane design enables the sensor to capture subtle deformation cues under small contact forces.

The remainder of this paper is organized as follows. Section 2 presents the sensor design and the results of sensor validation experiments, including the EndoTac architecture and optical design, geometric unwarping, low‐force sensitivity characterization, force estimation, and vessel palpation validation. Section 3 discusses the main findings and clinical translation considerations. Section 4 concludes the article and summarizes key findings, and Section 5 summarizes the hardware fabrication, calibration procedure, and experimental protocols used in this study.

## 2 Results

In this section, we evaluate the performance of the proposed EndoTac sensor and discuss its suitability for minimally invasive surgical applications. As illustrated in Figure 1a, EndoTac adopts a miniaturized design that enables the sensor to pass through a standard 12 mm trocar while maintaining a compact side‐facing sensing structure.

This compact form factor further allows the sensor to be manipulated by laparoscopic forceps in a manner similar to an ultrasound probe to scan along vascular structures. Inserted through a standard trocar, the sensor can be positioned onto the vessel surface and swept along its length under light, controlled contact. In this way, it functions as a tactile scanning probe, enabling sequential measurement of force and vessel deformation across different locations. This mode of operation is well suited to minimally invasive vascular palpation, where continuous exploration of delicate anatomy is often more informative than isolated point measurements.

We first introduce the structural design and optical configuration of the sensor, followed by the geometric unwarping method used to correct the distortion introduced by the cylindrical convex mirror. We then present vessel palpation experiments, as shown in Figure 1b, to evaluate the ability of the sensor to estimate vascular deformation from tactile images using a learning‐based model. Finally, we report experimental results that characterize the low‐force pixel‐level response of the sensor and its capability for force estimation. Together, these results demonstrate that EndoTac can effectively capture subtle deformation patterns and detect small interaction forces while maintaining a compact form factor suitable for laparoscopic procedures. A visual overview of the EndoTac hardware design, experimental setup, and sensor validation is provided in Video S1.

### 2.1 Design Overview

**FIGURE 2:**
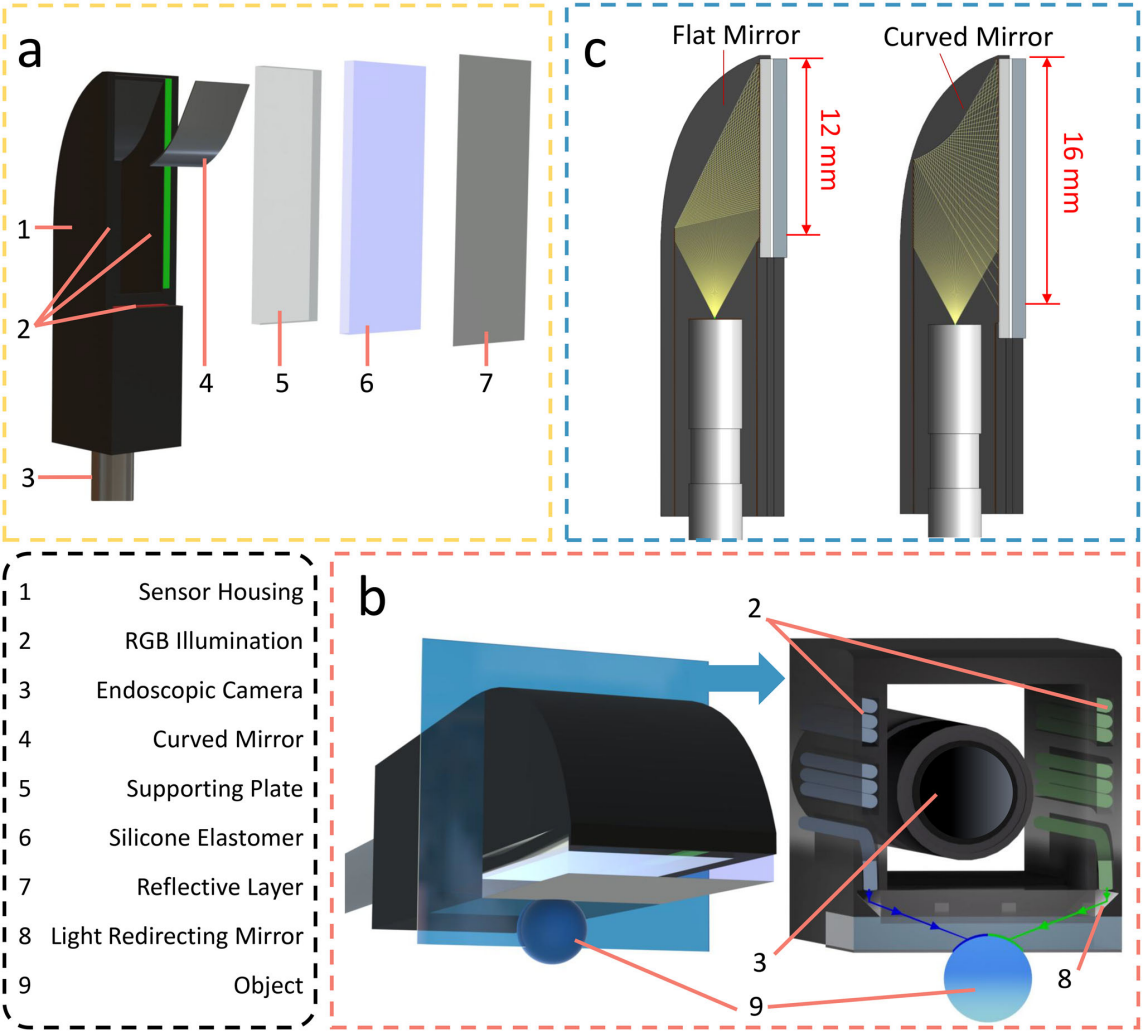
Design overview of the EndoTac sensor and its optical configuration. (a) Exploded view of the EndoTac sensor. (b) Cross‐sectional schematic of the sensing principle and illumination layout. Light from the side‐mounted optical fibers is redirected toward the sensing region, while the endoscopic camera captures the deformation of the elastomer surface through the convex mirror during contact with the vessel. (c) Light‐path comparison between flat‐mirror and convex‐mirror designs. The cylindrical convex mirror increases the effective sensing area from 12 by 6 mm to 16 by 6 mm with an increase of 33%, while maintaining the same overall form‐factor dimensions of the sensor.

EndoTac is designed with a miniaturized optical architecture that enables the sensor to fit within a standard 12 mm trocar while maintaining a side‐facing tactile sensing surface. A side‐facing configuration is particularly advantageous in MIS, where soft tissues and vessels are often manipulated using the lateral side of an instrument rather than by direct end‐on contact. This allows the sensor to be used in a probe‐like or sweeping manner for tissue exploration and palpation, while remaining compatible with common surgical motions. In addition, this overall architecture provides a practical foundation for further miniaturization and future integration into surgical grippers or other articulated MIS instruments. As illustrated in Figure 2a, the sensor integrates a compact endoscopic camera (Ezon Electronics, ϕ4 mm endoscopic camera), fiber‐based RGB illumination, a cylindrical convex mirror, a supporting plate, and a soft reflective elastomer membrane within a slender probe body. The convex mirror has a one‐dimensional curved profile along the longitudinal imaging direction and is approximately constant along the transverse direction of the sensing membrane. In the side‐view optical model, the mirror consists of a circular curved section connected to a terminal flat section.

The working principle of the sensor is shown in Figure 2b. The elastomer membrane is illuminated by optical fibers arranged along the sides of the probe. A thin strip of light‐redirecting mirror is positioned beneath the RGB LEDs at approximately $45^{\circ }$ to the light emitted from the optical fiber, so that the emitted light is reflected through $90^{\circ }$ before entering the elastomer layer, allowing the light to exit parallel to the sensing surface and improving illumination uniformity within the confined probe geometry. Otherwise, if the light is directed straight toward the elastomer, it would produce overexposed regions near the boundary of the sensing area and underexposed regions at the centre of the elastomer, resulting in unevenly distributed illumination. During contact, deformation of the reflective elastomer surface changes the shading pattern under RGB illumination color channels, which is captured by the endoscopic camera through the cylindrical convex mirror and used to encode tactile information.

To maximize sensing coverage within the limited probe size, EndoTac adopts a cylindrical convex mirror rather than a conventional flat mirror used in Gelsight wedge [25]. Unlike a flat mirror, which mainly redirects light and preserves a relatively narrow field of view, a convex mirror additionally diverges the reflected rays, allowing the camera to observe a wider portion of the sensing surface within the same optical geometry. As shown in Figure 2c, the convex mirror increases the observable sensing length from 12 to 16 mm while maintaining the same overall sensor form factor. Since the sensing width remains 6 mm, this expands the effective sensing area from $12\times 6\;{\mathrm{mm}}^{2}$ to $16\times 6\;{\mathrm{mm}}^{2}$, corresponding to a 33% increase in sensing area without increasing the probe cross‐section.

The mirror geometry is described by the terminal mirror angle α, the radius of curvature of the curved section r and d denotes the length of the orthogonal projection of the terminal flat mirror segment onto the sensing surface, as defined in Appendix (Section 1.1 Mirror Geometry Analysis, Appendix, Supporting Information). The selected geometry was

(1) $$\left[\alpha ,r,d\right]=\left[30^{\circ },\;6.44\;\mathrm{mm},\;6.51\;\mathrm{mm}\right].$$

Table 1 compares the cross‐sectional area and sensing area of representative miniature vision‐based tactile sensors. Using the ratio between sensor sensing area (SA) and cross‐sectional area (CA), i.e., SA/CA, as a measure of relative sensing coverage, EndoTac achieves the highest value among the compared sensors, demonstrating its ability to maintain a wide sensing region within a compact trocar compatible design. Importantly, this optical architecture is scalable. Further miniaturization could in principle be achieved by adopting a smaller camera module and proportionally reducing the dimensions of the surrounding optical and mechanical components. Under such geometric scaling, the absolute sensing area would decrease, but the relative sensing coverage, reflected by the SA/CA ratio, could in principle be preserved. This suggests that the proposed design is not only compact in its current form, but also provides a practical foundation for future downscaling without fundamentally compromising sensing efficiency.

**TABLE 1:** Comparison of miniaturized vision‐based tactile sensors.

Sensor	Cross‐sectional area [CA, ${\mathrm{mm}}^{2}$]	Sensing area [SA, ${\mathrm{mm}}^{2}$]	SA/ CA
GelSlim 3.0 [26]	80×37	675	0.23
CompVision [27]	11×10	5×5	0.23
GelSight‐mini [28]	32×28	19×15	0.32
TacScope [29]	$\pi \times 3.5^{2}$	$\pi \times 2.5^{2}$	0.51
DIGIT [30]	20×27	19×16	0.56
GelStereo 2.0 [31]	30×14	23×23	0.59
MiniTac [4]	$\pi \times 4^{2}$	$\pi \times 3.5^{2}$	0.77
DIGIT‐pinky [32]	$\pi \times 7.5^{2}$	181	1.02
**EndoTac (Ours)**	8×8	16×6	**1.5**

### 2.2 Image Unwarping

**FIGURE 3:**
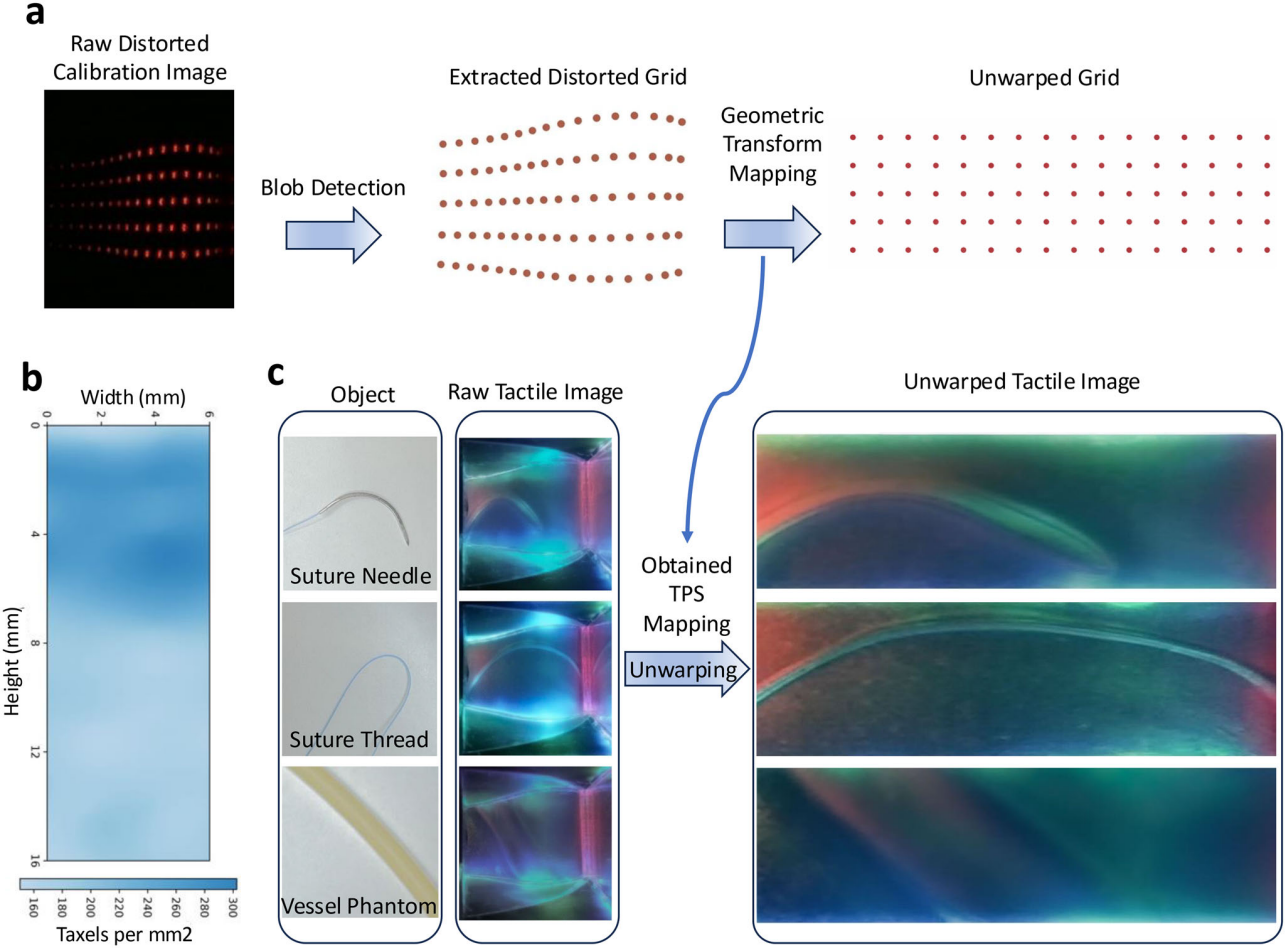
Geometric unwarping of distorted tactile images captured through the cylindrical convex mirror. (a) Calibration procedure for estimating the geometric transformation. A distorted uncalibrated image is processed using blob detection to extract warped grid points, which are then mapped to a planar grid through geometric transform mapping using a Thin Plate Spline (TPS) transformation. (b)Taxel density map across the sensing membrane. (c) Example tactile observations before and after unwarping. Raw tactile images captured by the camera through the convex mirror are transformed using the geometric transform mapping to recover the planar sensing surface, producing undistorted tactile representations of contacted objects.

The cylindrical convex mirror used in EndoTac enables a larger observable sensing region, but it also introduces geometric distortion in the captured tactile images. In addition, the mirror produces a nonuniform mapping across the sensing membrane. This mapping nonuniformity means that the membrane does not have perfectly uniform spatial resolution; instead, it contains regions with higher and lower effective spatial sampling density. The convex mirror therefore increases the observable sensing area at the cost of local spatial resolution. This mapping nonuniformity is not unique to the convex mirror, since a flat mirror also produces a nonlinear ray‐to‐surface mapping, however, the convex mirror deliberately redistributes the available camera field of view over a larger sensing region. This trade‐off was considered and discussed in the Appendix (Section 1.1 Mirror Geometry Analysis, Appendix, Supporting Information), where the peak angular to spatial mapping scale was used to select the mirror geometry. To further clarify this effect, we added a spatial‐resolution map showing the estimated taxel density per ${\mathrm{mm}}^{2}$ across the sensing area [33]. The heatmap shows that the effective spatial resolution varies across the membrane shown in Figure 3b. As a result, the raw tactile image does not directly correspond to the true planar geometry of the sensing surface. To recover an intuitive representation of the tactile contact, a geometric unwarping procedure is applied, as illustrated in Figure 3. In Figure 3a, a distorted calibration image is first captured through the sensor, and blob detection is used to extract the warped grid points. These distorted points are then associated with their corresponding positions on a planar grid, allowing a continuous pixel to pixel mapping obtained by Thin Plate Spline (TPS) interpolation, detailed in Section 5.2.

Once the mapping is computed, it is applied to subsequent tactile images to reconstruct the planar sensing surface. As shown in Figure 3c, the obtained mapping transforms the raw tactile images into unwarped tactile images that provide a more intuitive visualization of the contact geometry. Examples are shown for a suture needle, suture thread, and vessel phantom, which are commonly used in vascular surgical operations. These examples further demonstrate that the sensor can capture thin and flexible surgical objects, such as a suture needle and suture thread, as well as compliant objects, such as an artery phantom. Compared with the raw images, the corrected tactile images more clearly represent the shape and orientation of the contacted objects, facilitating interpretation of contact patterns and surface deformation during interaction.

### 2.3 Vessel Palpation

**FIGURE 4:**
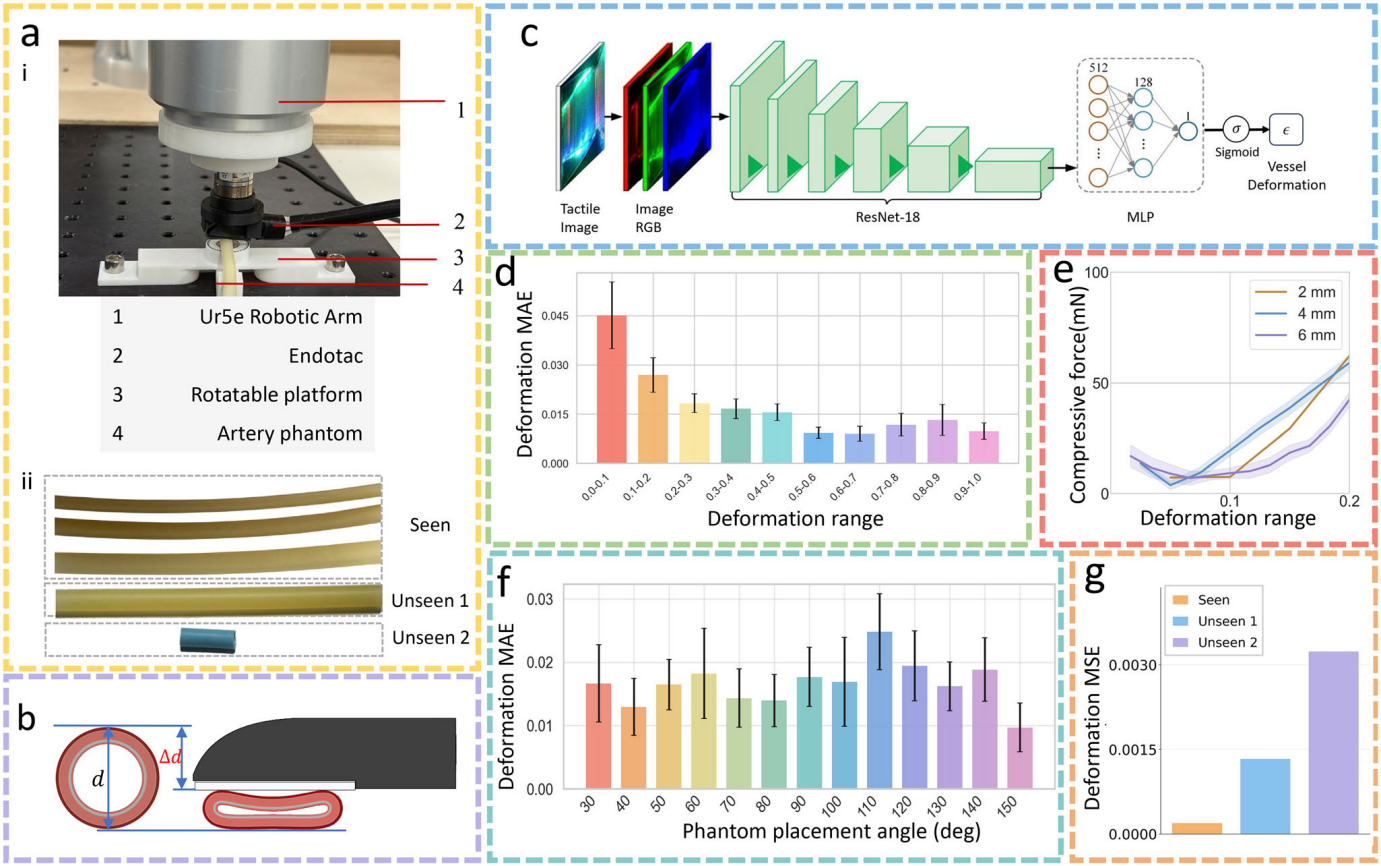
Evaluation of vessel deformation estimation using the EndoTac sensor during palpation experiments. (a) Experimental setup. (i) The EndoTac sensor mounted on a UR5e robotic arm presses against an artery phantom placed on a rotatable platform. (ii) Artery phantoms with different diameters used in the experiments. (b) Definition of vessel deformation. The deformation is defined as the relative change in vessel diameter [34]. (c) Learning pipeline for vessel deformation estimation. (d) Mean deformation error across different deformation ranges. (e) Compressive force required to achieve different vessel deformation ranges for artery phantoms with diameters of 2, 4, and 6 mm. (f) Mean vessel deformation estimation error across different vessel orientations (30
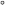
–150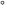). (g) Generalization performance of the deformation‐estimation model on the seen vessel test set and two independent unseen vessel phantom test sets.

To evaluate the ability of EndoTac to estimate vessel deformation during palpation and to assess its generalizability to unseen vessel conditions, experiments were conducted using both seen and unseen vessel phantoms. As shown in Figure 4a, the seen vessel set consisted of commercial artery phantoms with diameters of 2, 4, and 6 mm. These phantoms were used for model development and held‐out testing. Two additional independent unseen artery test sets were also collected. Unseen 1 was an 8 mm commercial artery phantom with the same mechanical properties as the seen artery phantom, and Unseen 2 was a 6 mm molded vessel phantom fabricated using Smooth‐Sil 950. These unseen phantoms were excluded from training.

The estimation performance across different deformation ranges is summarized in Figure 4d. Overall, the model achieves low deformation estimation error across most of the evaluated range, indicating that EndoTac can reliably capture vessel deformation during palpation. The largest mean absolute error is observed in the lowest deformation range, particularly for deformation values below 0.2. To further interpret this regime, Figure 4e shows the compressive force required to reach different deformation levels for artery phantoms of different diameters. In this low‐deformation range, the required compressive force is mainly within 0–50 mN, which falls within the force range further evaluated in the sensitivity experiments in Section 2.4.

Figure 4f,g further evaluate the robustness and generalizability of the deformation estimation model under different vessel conditions. In Figure 4f, the mean deformation error remains relatively even across phantom placement angles from 30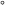 to 150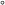, indicating stable performance over a wide range of vessel orientations. In Figure 4g, the model performance is further compared across the seen and unseen vessel test sets. The ResNet‐18 model achieved MSE values of $1.94\times 10^{-4}$, $1.33\times 10^{-3}$, and $3.24\times 10^{-3}$ on the seen, Unseen 1, and Unseen 2 test sets, respectively. Although the error increased on the unseen phantoms, the model maintained low deformation‐estimation error under changes in vessel diameter and material composition. These results show that the learned tactile image features can generalize beyond the vessel phantom conditions included during training.

To evaluate whether the deformation‐estimation pipeline is suitable for real‐time MIS palpation, inference speed was benchmarked on an NVIDIA GeForce RTX 4060 GPU. In an end‐to‐end benchmark including image loading, resizing, and inference, the ResNet‐18 model achieved 145.63 frames $s^{-1}$, corresponding to 6.87 ms per frame. This frame rate is above the endoscopic camera frame rate at 30 FPS, indicating that the ResNet‐18 deformation‐estimation pipeline can support real‐time vessel palpation.

### 2.4 Low‐Force Response and Force‐Estimation Performance

**FIGURE 5:**
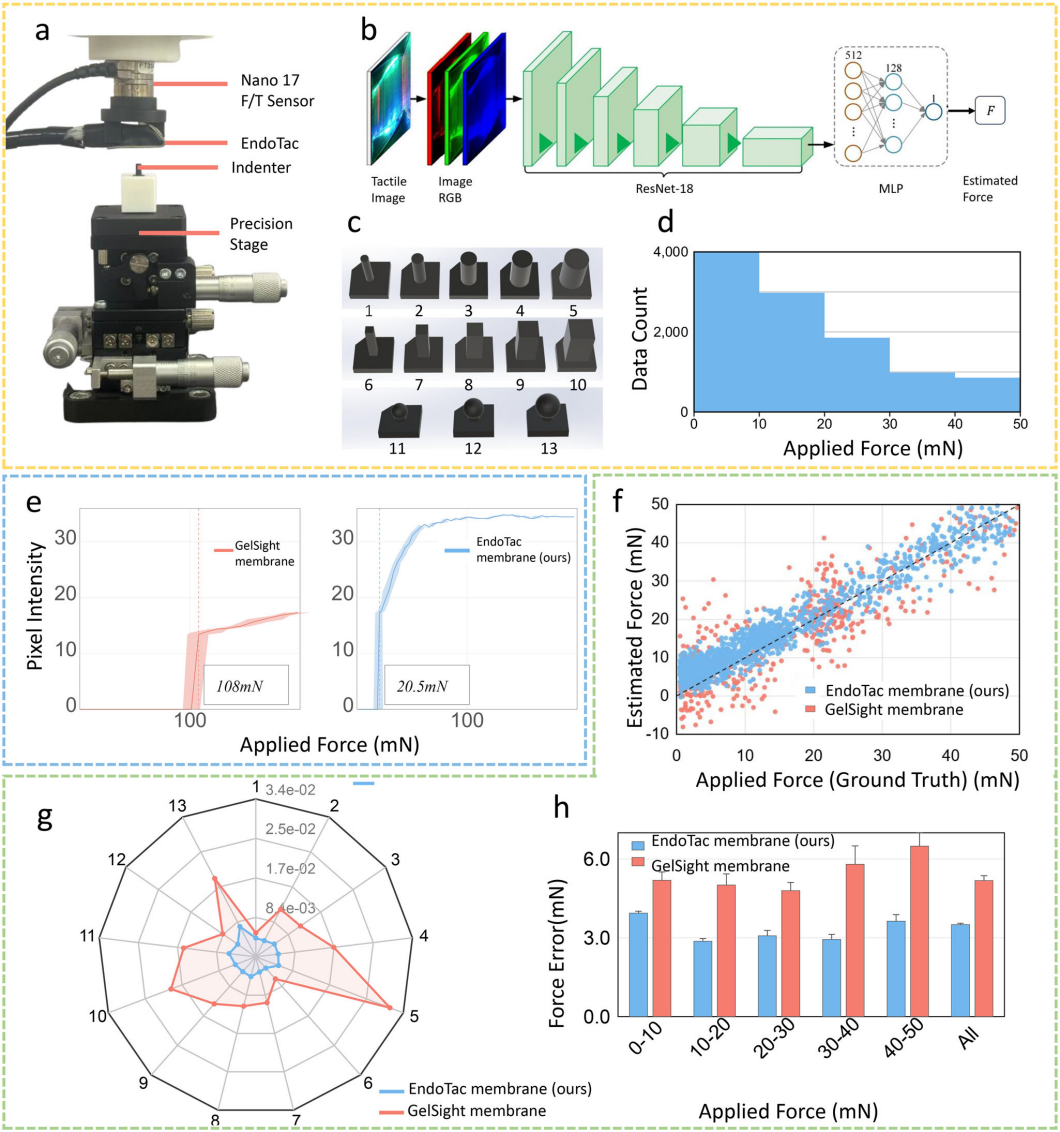
Low‐force response and force‐estimation performance of the EndoTac sensor through controlled indentation experiments. (a) Experimental setup for sensitivity experiment (b) Learning pipeline for force estimation. (c) Set of indenters with different geometries used during the experiments. The indenters include cylindrical tips (1–5: diameters 1, 1.5, 2, 2.5, and 3 mm), square tips (6–10: side lengths 1, 1.5, 2, 2.5, and 3 mm), and spherical tips (11–13: diameters 2, 2.5, and 3 mm). (d) Distribution of the collected dataset across the applied force range. (e) Comparison of average pixel intensity response between the proposed elastomer and the GelSight elastomer under increasing applied force. (f) Scatter plot of predicted force against ground truth force for the proposed elastomer and the GelSight elastomer. The dashed line indicates the ideal prediction (y=x). (g) Force estimation error across different indenter geometries. The radar plot summarizes the mean estimation error for each indenter used in the experiments, comparing the proposed elastomer with the GelSight elastomer baseline. (h) Mean absolute error (MAE) of force estimation across different force ranges.

Compared with many existing vision‐based tactile sensors, EndoTac adopts a type of material with lower shore hardness to fabricate the elastomer membranes. Because Shore hardness reflects resistance to indentation, a lower Shore hardness means the material deforms more easily under contact. Therefore, under the same applied pressure, the membrane can exhibit a larger deformation than conventional elastomers used in vision‐based tactile sensors, which improves the detectability of small‐force interactions. To characterize the low‐force sensing capability of the proposed membrane, two complementary analyses were performed. First, the direct pixel‐level response was evaluated by measuring the mean image‐intensity change under controlled indentation. This analysis was used to quantify the detection threshold, pixel‐level sensitivity slope, local linearity, and force resolution of the membrane. Second, learning‐based force estimation was evaluated to determine how effectively the tactile images support force prediction in the low‐force regime. To evaluate the performance of this membrane, the EndoTac elastomer was compared with the GelSight elastomer formulation, which is widely used in the vision‐based tactile sensing literature. The low‐force response and force‐estimation evaluation framework is summarized in Figure 5. As shown in Figure 5a, indentation experiments were conducted using a precision stage to provide controlled and repeatable loading, enabling fine indentation steps for low‐force response characterization. A Nano17 force–torque sensor was used to record the corresponding ground truth force. As shown in Figure 5c, a set of 13 indenters with different geometries was used to evaluate the robustness of the response across contact conditions. Following the experimental method introduced in [35] and subsequently adopted in later studies [36]. Force estimation was then performed using the learning pipeline shown in Figure 5b, where tactile images were processed by a ResNet‐18 backbone followed by an MLP regressor. As shown in Figure 5d, the lower sample density above 30 mN reflects the fact that the dataset was designed with emphasis on the low force regime, where low‐force tactile response and force‐estimation accuracy are most important. Importantly, this imbalance does not alter the central finding, because both methods were compared under the same data distribution and the proposed membrane remains superior across every force bin, including the higher‐force intervals. Additional baseline comparisons with a compact CNN [28] and a lightweight Vision Transformer [37] are provided in Appendix (Section 1.2 Baseline Model Comparison for Vessel Deformation Estimation, Appendix, Supporting Information) File, where ResNet‐18 showed the lowest overall deformation‐estimation error across the seen and unseen dataset.

#### 2.4.1 Pixel‐Level Low‐Force Response

To quantify the sensor response at the pixel level, the pixel intensity was used as a scalar measure of the visual deformation observed in the tactile image. The pixel‐level responses of the two membranes are compared in Figure 5e. The GelSight membrane begins to show a clear response only at around 107.9 mN, whereas the EndoTac membrane responds at a much smaller force of around 20.5 mN. The normalized pixel‐level sensitivity was quantified as the slope of the mean pixel‐intensity response with respect to applied force in the post‐trigger low‐force region [38, 39]. The EndoTac membrane was fitted over 20.5–50 mN, and the GelSight membrane was fitted over 107.9–150 mN. The EndoTac membrane achieved a normalized pixel‐level sensitivity slope of 0.501 with $R^{2}=0.982$, whereas the GelSight membrane achieved 0.038 with $R^{2}=0.949$. This corresponds to a higher normalized post‐trigger pixel‐level response slope for the EndoTac membrane. The fitted $R^{2}$ values indicate that both membranes exhibit a linear response within their respective fitted low‐force regions.

The force resolution was further estimated from the raw pixel‐intensity response using the baseline noise and fitted raw response slope. Using $F_{\mathrm{res}}=3\sigma_{I}/S_{I}$, where $\sigma_{I}$ is the standard deviation of the pre‐trigger raw pixel‐intensity response, and $S_{I}$ is the fitted raw response slope, the EndoTac membrane achieved an estimated force resolution of 23.97 mN, compared with 65.88 mN for the GelSight membrane. This indicates an improvement in force resolution for the proposed membrane. The more linear behavior of the GelSight membrane is associated with its higher effective stiffness. A stiffer elastomer has smaller incremental deformation, so the relationship between applied normal force and image change remains closer to proportional within the tested range. For the softer EndoTac membrane, larger deformation under the same load enhances the pixel‐level response, but also amplifies nonlinear effects arising from contact mechanics and material compliance, which can make the visual response less linear outside the fitted low‐force region.

The time‐dependent response of the proposed membrane was further evaluated using loading–unloading and constant‐hold tests. Similar loading–unloading, repeated loading, and temporal‐response characterizations have been used in prior MIS and soft tactile sensor studies to evaluate hysteresis, signal stability, and dynamic sensing behavior [4, 18, 21, 22, 24]. In the loading–unloading test, the membrane was indented from 0 to 0.6 mm and then unloaded back to 0 mm over five repeated cycles. The measured normal force were compared between loading and unloading at matched indentation depths. Hysteresis was quantified as the maximum loading–unloading difference normalized by the full‐scale loading response. The measured force hysteresis was 15.29%, indicating limited loading–unloading discrepancy within the tested indentation range. In the viscoelasticity test, the membrane was held at a fixed indentation depth for 60 s to assess short‐term relaxation behavior. During this hold, the measured force relaxed by 14.72%. This result indicates that the soft membrane exhibits measurable viscoelastic relaxation under sustained contact.

#### 2.4.2 Learning‐Based Force Estimation

The learning‐based force‐estimation performance for forces below 50 mN is shown in Figure 5f–h. In the scatter plot of Figure 5f, the predictions obtained using the EndoTac membrane are distributed more tightly around the linear line than those obtained using the GelSight membrane, indicating improved accuracy and consistency in force estimation. Figure 5g further compares the estimation error across the 13 indenter conditions, showing that the EndoTac membrane yields lower error for all indenters. Although the absolute error for indenter 13 is relatively larger than that of the other indenters, it remains small in magnitude, at less than $8\times 10^{-}3$ N. Therefore, the difference is limited in practical terms. Figure 5h further quantifies force estimation performance by plotting the MAE within successive normal force ranges from 0–10 to 40–50 mN, together with the overall error across the full 0–50 mN range. Across all bins, the proposed membrane consistently achieves lower MAE than the GelSight membrane, indicating that the performance improvement is maintained throughout the evaluated force range. The error bars denote the standard error of the mean, and their relatively small magnitude suggests that this improvement is consistent across repeated samples.

Overall, these results demonstrate that the softer EndoTac elastomer provides a clear advantage over the GelSight elastomer in the low‐force regime. The proposed membrane exhibits a lower detection threshold, a higher normalized pixel‐level sensitivity slope, strong local linearity within the fitted low‐force region, and improved raw‐noise‐based force resolution. These pixel‐level advantages are consistent with the learning‐based results, where the EndoTac membrane provides more accurate and robust force estimation across different indenters and force ranges.

## 3 Discussion

### 3.1 Positioning of EndoTac Among MIS Tactile Sensors

In the context of concurrent literature, the main contribution of EndoTac is not simply miniaturization, but the combination of enhanced low‐force response and relatively large sensing coverage within a trocar‐compatible vision‐based tactile sensor. Compared with existing MIS tactile sensors based on FBG sensing [40], electrical sensing [41], rolling indentation [5], robotic force mapping [42], or soft tactile skins [43], which are primarily designed to recover force or stiffness information, EndoTac provides image‐based access to local deformation and surface geometry in addition to force‐related cues.

### 3.2 Vision‐Based Tactile Sensing for MIS

Meanwhile, vision‐based tactile sensors have emerged as a powerful modality for capturing rich contact information by observing the deformation of a soft elastomer using an internal camera with structured illumination [28, 30, 31, 44, 45, 46, 47, 48, 49, 50, 51, 52]. When a force is applied, the elastomer deforms, and these deformations are captured at high spatial resolution, enabling precise reconstruction of surface geometry, slip, and small force variations. Such sensors have gained significant traction in robotic manipulation, where tactile feedback is crucial for grasp stability and fine manipulation [33, 46, 53, 54, 55].

Compared with many force‐oriented MIS tactile sensors, this imaging‐based principle is particularly attractive because it can provide spatially distributed deformation information rather than only a scalar or low‐dimensional force signal. The closest miniature vision‐based comparisons are MiniTac and TacScope, which demonstrate that vision‐based tactile sensing can be reduced to surgically relevant dimensions through miniaturized camera modules [4, 29]. Relative to these designs, however, EndoTac places greater advantages on two properties that are particularly important for MIS palpation. First, the use of a softer elastomer membrane improves the visual response to small applied loads and therefore reduces the detection threshold, increases the pixel‐level response slope, improves force resolution, and supports more accurate force estimation in the low‐force regime. Secondly, the cylindrical convex mirror increases the observable sensing region and thus improves relative sensing area efficiency within the same compact form factor. In addition, vessel deformation is clinically relevant beyond immediate intraoperative handling, since vascular deformation‐related biomechanical markers, including wall strain, wall shear stress, and plaque structural stress, have been associated with atherosclerotic plaque development, plaque vulnerability, and rupture risk (TLDR: This article summarizes studies conducted by collaborative laboratories on predictive biomechanical modeling of coronary plaques to give insights into the role of biomechanics in the development and localization of atherosclerosis, the morphologic features that determine vulnerable plaque stability, and emerging in vivo imaging techniques that may detect and characterize vulnerable plaque. see ref. [56]; TLDR: The current 'state of the art' on the interface between mechanical forces and atherosclerotic plaque biology is summarized and potential clinical applications and key questions for future research are identified. see ref. [57]; TLDR: Current experimental and clinical data linking PSS to the natural history of coronary artery disease, and potential for refining treatment options and predicting future events are discussed. see ref. [58]; TLDR: It is demonstrated that both MWS and WSS significantly correlate with atherosclerotic plaque initiation and development, and the association of MWS with coronary atherosclerosis, individually and combined with WSS is investigated. see ref. [59]). These considerations highlight the importance of detecting mechanically unsafe vessel behavior during surgical manipulation.

### 3.3 Comparison with Non‐Vision‐Based MIS Tactile Systems

The broader landscape of MIS tactile sensing further clarifies this trade‐off. To reduce the reliance on indirect visual inference and to provide surgeons with more direct interaction cues, equipping surgical tools with tactile sensing capabilities has attracted significant research attention, with the aim of restoring tactile information during minimally invasive procedures. Various sensing principles have been explored to address this challenge. Optical fiber–based force sensors, especially those that use Fiber Bragg Grating (FBG) technology, enable high‐resolution 3D contact force estimation while offering advantages such as electromagnetic immunity and sterilization compatibility. For example, Dong et al. [60] demonstrated a miniature triaxial FBG sensor tailored for MIS applications with reduced assembly complexity and improved isotropic sensitivity. Other FBG‐based MIS tactile systems have also been developed for miniature tissue palpation, laparoscopic grasping‐force feedback, and flexible endoscopic surgical robots [20, 61, 62]. These systems demonstrate the practicality of compact and electromagnetically robust force sensing in MIS, but their outputs are mainly force‐ or stiffness‐related rather than direct measurements of surface geometry or spatially distributed deformation. Recent non‐vision‐based and multimodal tactile systems further illustrate this direction. Hou et al. [18] integrated a piezoresistive three‐axis Micro‐Electro‐Mechanical Systems (MEMS) tactile sensor into gastrointestinal endoscopic forceps for online force feedback, while highly integrated MEMS force‐sensing modules have also been proposed for robot‐assisted MIS [63].

Electrical‐based force sensors have also been integrated into minimally invasive palpation tools to enable direct stiffness measurement in real time. For example, in [41], a catheter‐based device capable of mapping tissue stiffness was developed for intraoperative operations. Additionally, soft robotic skins composed of arrays of tactile elements have been proposed to cover larger organ surfaces in a single step; Campisano et al. introduced a deployable soft skin capable of differentiating lumps within ex vivo tissue by expanding fluidic chambers and recording local stiffness variations [43]. Capacitive and electrical forceps‐integrated sensors have been used to measure grasping, pulling, and multi‐axis tool–tissue forces [19, 64]. Other approaches combine force and angle measurements to estimate tissue stiffness and detect lumps, move the sensing structure away from the jaws to preserve grasper functionality, or use hybrid piezoresistive–optical sensing for stiffness measurement and tissue discontinuity detection [21, 22, 23]. These systems provide important force, stiffness, thickness, discontinuity, and multidimensional force information, but they are generally designed around local tool–tissue force or stiffness measurement rather than wide area deformation imaging.

Soft and fluidic sensing structures provide another route toward compliant tool–tissue interaction and larger contact coverage. For example, multichannel soft microfluidic force sensors have recently been integrated with laparoscopic graspers to provide compliant force measurements during grasping tasks [24]. More broadly, recent work on flexible pressure tactile sensors and liquid‐metal‐based 3D tactile sensing has highlighted advances in sensitivity, linear range, multidimensional force sensing, miniaturization, and data‐driven dynamic force estimation [65, 66]. These systems provide important force, stiffness, thickness, discontinuity and multidimensional force information, but they are generally designed around local tool–tissue force or stiffness measurement rather than wide area deformation imaging.

Haptic‐feedback approaches have also been investigated to return force or tactile information to the surgeon during MIS and robot‐assisted MIS [1, 2]. These systems are important for improving surgical perception and tool–tissue interaction, but they still require reliable sensing information from the surgical tool or environment. Therefore, compact tactile sensing remains an important enabling component for clinically useful haptic feedback.

### 3.4 Miniaturization and Sensing Coverage for Vessel Palpation

Representative designs of vision–based tactile sensors include GelSight‐type reflective elastomer sensors, TacTip‐style marker‐based sensors, compact DIGIT‐type sensors, omnidirectional GelTip sensors, and mobile or rotating tactile sensors [30, 45, 46, 53, 67, 68, 69, 70, 71]. These developments demonstrate the versatility of vision‐based tactile sensing, but most were designed for general robotic manipulation rather than the dimensional and deployment constraints of MIS.

Recent miniaturization efforts have reduced sensor size through compact RGB illumination layouts, thin optical paths, mirror‐assisted imaging, remote imaging using fiber bundles, and optimization‐based optical layouts [25, 26, 30, 32, 72, 73, 74]. These approaches show that vision‐based tactile sensing can be made more compact, but most have been developed for general robotic manipulation rather than MIS‐compatible deployment. Relatively few studies have specifically addressed miniaturization and integration under surgical constraints in terms of trocar compatibility.

Despite their success in robotic manipulation, vision‐based tactile sensors have seen limited adoption in MIS because conventional designs often require optical paths, cameras, and illumination units that are difficult to miniaturize into a trocar compatible size. Most existing designs remain too bulky to pass through standard MIS trocars with diameters up to 12 mm [26, 28, 53, 72]. To address this, recent studies have aimed to design vision‐based tactile sensors specifically for MIS. For instance, the MiniTac sensor achieved an 8 mm diameter, intended for integration with the da Vinci surgical robotic system [4]. More recently, TacScope further reduced the diameter of the vision‐based tactile sensor to 5 mm [29]. These MIS‐oriented sensors represent important progress toward trocar‐compatible vision‐based tactile sensing. However, miniaturization alone does not fully address the sensing requirements of vessel palpation, where the sensor must also preserve sufficient contact area and sensitivity to capture distributed deformation along the vessel wall.

The vision‐based tactile sensors developed recently that meet trocar size constraints often do so at the expense of sensing areas. For example, MiniTac [4] and TacScope [29] can traverse standard trocars; however, their sensing areas are confined to the limited front plane of the laparoscopic tool tip.

This tip‐localized configuration limits the observable sensing region and therefore the amount of spatial deformation information that can be captured during extended tissue contact.

In addition, these sensors typically employ silicone membranes originally developed for vision‐based tactile sensing in general robotic manipulation [28], where durability against repeated contact with rigid objects is prioritized over sensitivity [75]. As a result, such sensors are less suitable for detecting the subtle deformations associated with gentle interactions with fragile vascular tissue.

Therefore, beyond reducing sensor diameter, an MIS tactile sensor for vessel palpation should also consider sensing coverage, membrane sensitivity, and the sensing area/cross‐sectional area ratio within the available trocar‐compatible dimension.

Based on this comparison, EndoTac is positioned as a complementary approach rather than a replacement for local force or stiffness sensing. Compared with forceps‐integrated MEMS, capacitive, FBG, off‐the‐jaw, soft microfluidic, and haptic‐feedback systems, EndoTac prioritizes spatially distributed deformation imaging over a side‐facing membrane. Compared with deployable skins or scanning palpation probes, EndoTac aims to provide an extended sensing region within a compact, self‐contained, trocar‐compatible probe geometry, without requiring the sensor to unfold over the tissue or repeatedly scan to build a deformation map. These trade‐offs emphasize side‐facing sensing coverage and deformation imaging for vessel palpation.

### 3.5 Scalability and Instrument‐Level Integration

The proposed optical design also provides a clear route toward further miniaturization and broader surgical deployment. Specifically, the combination of a cylindrical convex mirror and a side facing sensing surface is not only effective in the current prototype, but also scalable, since the overall geometry can in principle be reduced proportionally with a smaller camera module while preserving a similar relative sensing area. Therefore, the convex mirror layout offers a practical optical foundation for maintaining tactile coverage even as the sensor diameter is reduced further. At the same time, the side facing sensing configuration is suited for integration into surgical grippers, where the lateral surfaces of the tool are frequently used for tissue manipulation. Together, these features suggest that the proposed design is not limited to a single probe embodiment, but may support a wider range of future MIS applications through further miniaturization and instrument level integration.

### 3.6 Sensor Response Characteristics

The working range of the tactile response could potentially be increased by investigating new membrane materials and structures that can respond to low‐force soft tissue interaction while also tolerating larger applied forces. In the current prototype, a soft elastomer was selected because it deforms more readily under gentle contact, which is important for detecting higher deformation during soft‐tissue palpation. Future work could explore elastomers or composite membrane designs with a broader usable deformation range, allowing the sensor to preserve low‐force sensitivity while extending the measurable force range.

The viscoelasticity experiment also demonstrated measurable viscoelastic relaxation of the soft membrane under sustained contact. However, this test represents a conservative sustained‐contact condition, since the membrane was held against a rigid indenter for 60 s. In the intended EndoTac vessel‐palpation scenario, contact is typically made with compliant soft tissue and involves brief scanning or palpation rather than prolonged rigid indentation. Therefore, the measured relaxation characterizes the time‐dependent behavior of the soft membrane under a worst‐case sustained‐contact condition, while the effect is expected to be less critical during short‐duration soft‐tissue palpation.

### 3.7 Clinical Translation

For future clinical translation, several practical considerations should also be addressed. The modular structure of EndoTac provides a potential route for sterilization and clinical deployment, since the contact‐facing sensing probe can be detached from the camera body and treated as a disposable sterile consumable. This would reduce the need to repeatedly sterilize the soft tactile membrane, while the reusable imaging component could be implemented using clinically used endoscopic camera hardware compatible with established sterilization or sterile‐barrier workflows. In terms of robustness and membrane durability, EndoTac is designed primarily for gentle interaction with soft tissue rather than repeated high‐load contact with rigid objects. Therefore, a disposable sensing probe design can reduce concerns related to cumulative membrane wear, coating degradation, or long‐term mechanical fatigue. Realistic surgical environments may also involve wet tissue surfaces, blood contamination, and incidental contact with surrounding tissues, which can introduce optical occlusion, alter surface reflectance, and generate unexpected deformation patterns. These factors highlight the need for improved probe sealing, waterproofing, and model robustness under intraoperative contact variability. Finally, because the current prototype is designed to pass through a standard trocar and uses a side‐facing sensing surface, it could be incorporated into laparoscopic workflows as an adjunct tactile palpation or scanning probe, depending on the specific surgical task.

## 4 Conclusion

This work presents EndoTac, a compact vision‐based tactile sensor for MIS that combines a low detection threshold, high pixel‐level response slope, improved force resolution, and accurate low‐force estimation with relatively large sensing coverage in a trocar‐compatible form factor. By integrating an endoscopic camera with a cylindrical convex mirror, the sensor enlarges the observable sensing region without increasing the sensor diameter, leading to a high relative sensing area within a miniaturized design. This is particularly important for MIS, where a larger sensing region allows soft tissues deforming over larger areas to be captured during palpation and vessel interaction, rather than restricting sensing to a small point at the probe tip.

In addition to the optical design, EndoTac employs a soft elastomer membrane to improve low‐force tactile response and force‐estimation performance. Experimental results show that the proposed membrane provides a stronger visual response to small applied loads, achieves a detection threshold of 20.5 mN, a normalized pixel‐level sensitivity slope of 0.501 a.u./mN, and an estimated force resolution of 23.97 mN, and supports more accurate force estimation below 50 mN than the GelSight elastomer baseline. Vessel palpation experiments further demonstrate that the sensor can estimate vascular deformation with high accuracy across different phantom sizes and orientations.

Overall, EndoTac shows that it is possible to combine miniaturization, large relative sensing area, and strong low‐force tactile response, improved force resolution, and accurate low‐force estimation within a single vision‐based tactile sensor for MIS. These results provide a practical foundation for future tactile sensing tools that can deliver richer and more intuitive mechanical feedback during minimally invasive surgical procedures.

## 5 Method

This section describes the hardware implementation, calibration procedure, and experimental protocols used to evaluate the EndoTac sensor. We first describe the integration of the sensing components and fabrication of the tactile membrane. Next, the geometric calibration procedure used to correct mirror‐induced image distortion is introduced. Finally, the experimental protocols for sensitivity testing and vessel palpation are presented together with the data collection and model training procedures used for force and deformation estimation.

### 5.1 Sensor Design and Fabrication

The EndoTac sensor integrates an endoscopic camera, RGB illumination, a cylindrical convex mirror, and a deformable elastomer membrane within a compact housing designed for minimally invasive surgical tools.

1. A ϕ4 mm endoscopic camera is used as the imaging module. The camera captures the deformation of the elastomer membrane through a cylindrical convex mirror, which redirects the optical path and allows the sensing surface to be positioned along the side of the probe.
2. Illumination is provided using end‐glowing optical fibers with a diameter of 0.25 mm. The fibers deliver RGB light to the sensing region and are arranged along the sides of the elastomer to produce directional illumination suitable for visually capturing surface deformation patterns.
3. The tactile membrane consists of a soft silicone elastomer base layer (Ecoflex 00‐31 Near Clear) coated with a thin reflective layer formed using silver silicone ink (Yonglihua Ink, silver silicone ink). The elastomer base layer is fabricated by mixing the silicone components at a 1:1 ratio and casting the material into a mold with a thickness of 1 mm. After curing, a reflective coating is applied using screen printing to create a uniform reflective surface that enables the camera to capture deformation patterns of the membrane.

### 5.2 Calibration and Unwarping

The cylindrical convex mirror used in the sensor introduces geometric distortion in the captured tactile images. To recover the original planar geometry of the sensing surface, a geometric unwarping procedure based on Thin Plate Spline (TPS) interpolation is applied.

During calibration, the sensor is placed above a flat display showing a regular grid of dots with known spatial coordinates. A calibration image is captured through the sensor, and blob detection is used to identify the distorted positions of the grid points in the tactile image.

Let the distorted pixel coordinates be denoted as ${\left\{x_{i}\right\}}_{i=1}^{N}\subset R^{2}$ and the corresponding reference planar grid coordinates be denoted as ${\left\{y_{i}\right\}}_{i=1}^{N}\subset R^{2}$. The TPS mapping $f:R^{2}\to R^{2}$ satisfies the interpolation condition

$$f\left(x_{i}\right)=y_{i},\;i=1,\text{…},N.$$

The mapping takes the form

$$f\left(x\right)=Ax+b+\sum_{i=1}^{N}w_{i}U(∥x-x_{i}∥)$$

where A is a 2×2 affine matrix, b is a translation vector, $w_{i}$ are TPS weights, and $U\left(r\right)=r^{2}\log r$ is the radial basis kernel.

The TPS parameters are obtained by solving the interpolation constraints while minimizing the bending energy of the transformation. Once the mapping is computed, it is applied to all subsequent tactile images to reconstruct the undistorted sensing surface.

### 5.3 Sensor Response Testing Protocol

Sensor response was evaluated through controlled indentation experiments using the setup shown in Figure 5a. The EndoTac sensor was mounted together with an ATI Nano17 force/torque sensor, while the indenters were mounted on a precision stage with a displacement resolution of 0.01 mm to enable controlled and repeatable low‐force indentation. A set of indenters with different geometries was used, as shown in Figure 5c, including cylindrical tips (1–5: diameters 1, 1.5, 2, 2.5, and 3 mm), square tips (6–10: side lengths 1, 1.5, 2, 2.5, and 3 mm), and spherical tips (11–13: diameters 2, 2.5, and 3 mm). During each trial, the indenter was incrementally moved toward the sensor surface while tactile images and corresponding force measurements were recorded simultaneously.

To quantify the pixel‐level response of the sensor, the tactile image captured during contact was compared with a reference image obtained under no contact. For each pixel location (x,y), the absolute intensity difference between the tactile image I(x,y) and the reference image $I_{\mathrm{ref}}\left(x,y\right)$ was computed as

$$D\left(x,y\right)=|I\left(x,y\right)-I_{\mathrm{ref}}\left(x,y\right)|.$$

The pixel intensity value was then defined as the average of D(x,y) over all pixels in the image, providing a scalar measure of the visual response induced by elastomer deformation.

$$\text{pixel intensity}=\frac{1}{N}\sum_{\left(x,y\right)}D\left(x,y\right)$$

The detection threshold was defined as the first force level at which the mean pixel‐intensity response showed a clear increase from the pre‐trigger baseline. Pixel‐level sensitivity was quantified by fitting a linear regression to the post‐trigger low‐force region of the mean pixel‐intensity–force curve. The fitted slope was reported as the pixel‐level sensitivity, and the coefficient of determination $R^{2}$ was used to quantify local linearity within the fitted region. Force resolution was estimated from the raw pixel‐intensity response as

$$F_{\mathrm{res}}=\frac{3\sigma_{I}}{S_{I}},$$

where $\sigma_{I}$ is the standard deviation of the pre‐trigger raw pixel‐intensity response and $S_{I}$ is the fitted raw response slope in the post‐trigger region.

More than 10,000 image–force pairs were collected for each elastomer formulation. The distribution of the collected samples across the applied‐force range is shown in Figure 5d. For each elastomer dataset, 6000 samples were used for training and 1500 samples were held out for validation.

Force estimation was performed using the learning pipeline shown in Figure 5b. Tactile RGB images were processed by ResNet‐18 backbone followed by a multilayer perceptron (MLP) regression head. The input images were resized to 224×224 pixels. The model was trained using mean squared error (MSE) loss with the Adam optimizer, a batch size of 32, and 30 training epochs. The random seed was fixed at 42. Training and inference were performed using PyTorch on an NVIDIA GeForce RTX 4060 GPU.

Loading–unloading hysteresis and constant‐hold relaxation were evaluated following the general characterization approach used in previous tactile sensor studies, where loading–unloading curves, repeated loading, signal drift, and response stability are used to assess hysteresis, temporal stability, and dynamic sensing behavior [4, 18, 21, 22, 24, 65, 66]. Loading–unloading hysteresis was evaluated using a step‐and‐hold indentation protocol. The membrane was indented from 0 to 0.6 mm in 0.1 mm increments and then unloaded back to 0 mm using the same step size. The test was repeated for five cycles. At each indentation step, the robot was held at the target position while tactile images, indentation depth, and force measurements were recorded synchronously. The pixel‐level response was calculated using the same image‐difference metric defined above. For each matched indentation depth, the mean loading response and mean unloading response were compared. Pixel‐response hysteresis was calculated as

(2) $$H_{I}=\frac{\max_{d}\left|I_{\mathrm{loading}}\left(d\right)-I_{\mathrm{unloading}}\left(d\right)\right|}{I_{\mathrm{FS}}}\times 100\%,$$

where $I_{\mathrm{loading}}\left(d\right)$ and $I_{\mathrm{unloading}}\left(d\right)$ are the pixel‐level responses during loading and unloading at indentation depth d, and $I_{\mathrm{FS}}$ is the full‐scale loading response. Force hysteresis was calculated using the same form by replacing the pixel‐level response with the measured normal force.

Short‐term viscoelastic relaxation was evaluated using a 60 s constant‐indentation hold. During this test, the indentation depth was held constant while the force and pixel‐level response were recorded continuously. The force relaxation was calculated as

(3) $$R_{F}=\frac{F_{\mathrm{initial}}-F_{\mathrm{final}}}{F_{\mathrm{initial}}}\times 100\%,$$

where $F_{\mathrm{initial}}$ is the mean force during the first 1 s of the hold and $F_{\mathrm{final}}$ is the mean force during the final 5 s of the hold. The pixel‐response drift was calculated similarly using the initial and final pixel‐level responses. Because the current constant‐hold test was performed as a single trial, the relaxation and drift values are reported as single‐trial measurements rather than mean ± standard deviation.

### 5.4 Artery Phantom Palpation Protocol

Vessel palpation experiments were conducted using silicone artery phantoms (Limbs & Things vascular vessels artery) with diameters of 2, 4 and 6 mm as seen tissue and artery phantoms with 8 and 6 mm artery phantoms molded with Smooth‐Sil 950 as unseen tissue, as shown in Figure 4a–ii. The experimental setup is illustrated in Figure 4ai, where the EndoTac sensor was mounted on a UR5e robotic arm and pressed against the artery phantom positioned on a rotatable platform. During indentation, the corresponding compressive force was recorded using an ATI Nano17 force/torque sensor. To generate diverse contact conditions, the platform allowed the vessel orientation angle to vary from 30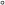 to 150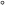 in 10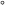 increments. For each angle, five indentation trials were performed while tactile images were captured at deformation increments of 0.1 mm.

In total, 7800 tactile image and deformation pairs were collected. The dataset was randomly split into 80% training data and 20% testing data. Vessel deformation was defined as the relative change in vessel diameter, which is defined as vascular strain as a significant measurement of vascular shape and condition proposed by de Kort et al. [34], as illustrated in Figure 4b, where d denotes the original vessel diameter and Δd denotes the indentation depth produced during contact. The deformation value was therefore calculated as Δd/d.

The deformation estimation model adopted the learning pipeline shown in Figure 4c. The same ResNet‐18 backbone used for force estimation was combined with an MLP head, and the output layer was modified with a sigmoid activation function to predict a normalized vessel deformation value directly from tactile images. The network was trained using MSE loss with the Adam optimizer, a learning rate of $1\times 10^{-4}$, a batch size of 32, and 30 epochs. During training, data augmentation was applied to the input images, including small random crop/zoom changes, small brightness, contrast, and saturation perturbations, and small affine transformations involving rotation, translation, and scale changes. Data augmentation was applied only to the training set and not to the validation, seen test, or independent unseen test sets. The random seed was fixed at 42. The independent test sets were used only for final evaluation. Training and inference were performed using PyTorch on an NVIDIA GeForce RTX 4060 GPU.

## Conflicts of Interest

The authors declare no conflicts of interest.

## Supporting information


**Supporting File 1**: advs76921‐sup‐0001‐SuppMat.pdf.

**Supporting File 2**: advs76921‐sup‐0002‐VideoS1.mp4.

## Data Availability

The data that supports the findings of this study are available in the supplementary material of this article.

## References

[1] E. Abdi , D. Kulić , and E. Croft , “Haptics in Teleoperated Medical Interventions: Force Measurement, Haptic Interfaces and Their Influence on User's Performance,” IEEE Transactions on Bio‐Medical Engineering 67, no. 12 (2020): 3438–3451. 32305890 10.1109/TBME.2020.2987603

[2] J.‐J. Cabibihan , A. Y. Alhaddad , T. Gulrez , and W. J. Yoon , “Influence of Visual and Haptic Feedback on the Detection of Threshold Forces in a Surgical Grasping Task,” IEEE Robotics and Automation Letters 6, no. 3 (2021): 5525–5532. 10.1016/j.dib.2022.108045 PMC8943423 35341034

[3] J. Marescaux and M. Diana , “Next Step in Minimally Invasive Surgery: Hybrid Image‐Guided Surgery,” Journal of Pediatric Surgery 50, no. 1 (2015): 30–36. 25598089 10.1016/j.jpedsurg.2014.10.022

[4] W. Li , Z. Zhao , L. Cui , et al., “MiniTac: An Ultra‐Compact 8 mm Vision‐Based Tactile Sensor for Enhanced Palpation in Robot‐Assisted Minimally Invasive Surgery,” IEEE Robotics and Automation Letters 9, no. 12 (2024): 11170–11177.

[5] H. Liu , J. Li , X. Song , L. D. Seneviratne , and K. Althoefer , “Rolling Indentation Probe for Tissue Abnormality Identification During Minimally Invasive Surgery,” IEEE Transactions on Robotics 27, no. 3 (2011): 450–460.

[6] D. Stoyanov , G. P. Mylonas , F. Deligianni , A. Darzi , and G. Z. Yang , “Soft‐Tissue Motion Tracking and Structure Estimation for Robotic Assisted MIS Procedures,” Medical Image Computing and Computer‐assisted Intervention: MICCAI … International Conference on Medical Image Computing and Computer‐assisted Intervention 8, no. Pt 2 (2005): 139–146, 10.1007/11566489_18. 16685953

[7] P. Mountney and G.‐Z. Yang , “Soft Tissue Tracking for Minimally Invasive Surgery: Learning Local Deformation Online,” Medical Image Computing and Computer‐assisted Intervention: MICCAI … International Conference on Medical Image Computing and Computer‐assisted Intervention 11, no. Pt 2 (2008): 364–372, 10.1007/978-3-540-85990-1_44. 18982626

[8] B. Lin , Y. Sun , X. Qian , D. Goldgof , R. Gitlin , and Y. You , “Video‐Based 3D Reconstruction, Laparoscope Localization and Deformation Recovery for Abdominal Minimally Invasive Surgery: A Survey,” The International Journal of Medical Robotics and Computer Assisted Surgery 12, no. 2 (2016): 158–178, 10.1002/rcs.1661. 25931190

[9] A. Chaturvedi , S. A. Shukair , P. Le Rolland , M. Vijayvergia , J. W. Gunn , and H. Subramanian , “Blood Vessel Detection, Localization and Estimation Using a Smart Laparoscopic Grasper: A Monte Carlo Study,” Biomedical Optics Express 9, no. 5 (2018): 2027–2040. 29760967 10.1364/BOE.9.002027 PMC5946768

[10] A. S. Sethi , S. M. Regan , and C. P. Sundaram , “The Use of a Doppler Ultrasound Probe During Vascular Dissection in Laparoscopic Renal Surgery,” Journal of Endourology 23, no. 9 (2009): 1377–1382. 19698037 10.1089/end.2009.0397

[11] N. Montaña‐Brown , J. Ramalhinho , M. Allam , B. Davidson , Y. Hu , and M. J. Clarkson , “Vessel Segmentation for Automatic Registration of Untracked Laparoscopic Ultrasound to CT of the Liver,” International Journal of Computer Assisted Radiology and Surgery 16, no. 7 (2021): 1151–1160. 34046826 10.1007/s11548-021-02400-6 PMC8260404

[12] D. Ragab , D. K. Saha , E. Rendon‐Morales , and H. Godaba , “Compact Planar Low‐Voltage Electroadhesion Pads for Reversible Tissue and Hydrogel Adhesion,” Advanced Materials Technologies 10, no. 23 (2025): e01189, 10.1002/admt.202501189.

[13] J. Colan , A. Davila , and Y. Hasegawa , “Tactile Feedback in Robot‐Assisted Minimally Invasive Surgery: A Systematic Review,” The International Journal of Medical Robotics and Computer Assisted Surgery 20, no. 6 (2024): e70019, 10.1002/rcs.70019. 39644216 PMC11624840

[14] A. H. Hadi Hosseinabadi and S. E. Salcudean , “Force Sensing in Robot‐Assisted Keyhole Endoscopy: A Systematic Survey,” The International Journal of Robotics Research 41, no. 2 (2022): 136–162, 10.1177/02783649211052067.

[15] N. Bandari , J. Dargahi , and M. Packirisamy , “Tactile Sensors for Minimally Invasive Surgery: A Review of the State‐of‐the‐Art, Applications, and Perspectives,” IEEE Access 8 (2020): 7682–7708, 10.1109/ACCESS.2019.2962636.

[16] W. Othman , Z.‐H. A. Lai , C. Abril , et al., “Tactile Sensing for Minimally Invasive Surgery: Conventional Methods and Potential Emerging Tactile Technologies,” Frontiers in Robotics and AI 8 (2022), 10.3389/frobt.2021.705662. PMC8777132 35071332

[17] J. Park , B. Seo , Y. Jeong , and I. Park , “A Review of Recent Advancements in Sensor‐Integrated Medical Tools,” Advanced Science 11, no. 20 (2024): 2307427, 10.1002/advs.202307427. 38460177 PMC11132050

[18] C. Hou , H. Gao , X. Yang , et al., “A Piezoresistive‐Based 3‐Axial MEMS Tactile Sensor and Integrated Surgical Forceps for Gastrointestinal Endoscopic Minimally Invasive Surgery,” Microsystems & Nanoengineering 10, no. 1 (2024): 141, 10.1038/s41378-024-00774-6. 39327456 PMC11427553

[19] U. Kim , D.‐H. Lee , W. J. Yoon , B. Hannaford , and H. R. Choi , “Force Sensor Integrated Surgical Forceps for Minimally Invasive Robotic surgery,” IEEE Transactions on Robotics 31, no. 5 (2015): 1214–1224, 10.1109/TRO.2015.2473515.

[20] P. Wang , S. Zhang , Z. Liu , et al., “Smart Laparoscopic Grasper Integrated with Fiber Bragg Grating Based Tactile Sensor for Real‐Time Force Feedback,” Journal of Biophotonics 15, no. 5 (2022): e202100331, 10.1002/jbio.202100331. 35020276

[21] W. Othman , K. E. Vandyck , C. Abril , et al., “Stiffness Assessment and Lump Detection in Minimally Invasive Surgery Using In‐House Developed Smart Laparoscopic Forceps,” IEEE Journal of Translational Engineering in Health and Medicine 10 (2022): 1–10, 10.1109/JTEHM.2022.3180937. PMC9216325 35774413

[22] W. Othman , K. E. Vandyck , M.‐S. Abdul‐Hamid , et al., “Off‐the‐Jaw Tactile Sensing System for Tissue Stiffness and Thickness Assessment in Minimally Invasive Surgery,” IEEE Access 13 (2025): 49320–49333, 10.1109/ACCESS.2025.3550948.

[23] N. M. Bandari , R. Ahmadi , A. Hooshiar , J. Dargahi , and M. Packirisamy , “Hybrid Piezoresistive‐optical Tactile Sensor for Simultaneous Measurement of Tissue Stiffness and Detection of Tissue Discontinuity in Robot‐Assisted Minimally Invasive Surgery,” Journal of Biomedical Optics 22, no. 7 (2017): 77002, 10.1117/1.JBO.22.7.077002. 28734117

[24] W. Othman and M. A. Qasaimeh , “Multichannel Soft Microfluidic Force Sensors: Design, Characterization, and Application in Laparoscopy,” Microsystems & Nanoengineering 12, no. 1 (2026): 138, 10.1038/s41378-026-01263-8. 42002568 PMC13092641

[25] S. Wang , Y. She , B. Romero , and E. Adelson , “GelSight Wedge: Measuring High‐Resolution 3D Contact Geometry with a Compact Robot Finger,” in 2021 IEEE International Conference on Robotics and Automation (ICRA) (IEEE, 2021), 6468–6475.

[26] I. H. Taylor , S. Dong , and A. Rodriguez , “GelSlim 3.0: High‐Resolution Measurement of Shape, Force and Slip in a Compact Tactile‐Sensing Finger,” in 2022 International Conference on Robotics and Automation (ICRA) (IEEE, 2022), 10781–10787.

[27] L. Luo , B. Zhang , Z. Peng , et al., “CompdVision: Combining near‐Field 3D Visual and Tactile Sensing Using a Compact Compound‐Eye Imaging System,” in 2024 IEEE/RSJ International Conference on Intelligent Robots and Systems (IROS) (IEEE, 2024), 262–268.

[28] W. Yuan , S. Dong , and E. H. Adelson , “GelSight: High‐Resolution Robot Tactile Sensors for Estimating Geometry and Force,” Sensors 17, no. 12 (2017): 2762, 10.3390/s17122762. 29186053 PMC5751610

[29] M. R. I. Prince , S. Athar , P. Zhou , and Y. She , “TacScope: A Miniaturized Vision‐Based Tactile Sensor for Surgical Applications,” Advanced Robotics Research (2025): e202500117.

[30] M. Lambeta , P.‐W. Chou , S. Tian , et al., “DIGIT: A Novel Design for a Low‐Cost Compact High‐Resolution Tactile Sensor with Application to In‐Hand Manipulation,” IEEE Robotics and Automation Letters 5, no. 3 (2020): 3838–3845.

[31] C. Zhang , S. Cui , S. Wang , et al., “GelStereo 2.0: An Improved GelStereo Sensor with Multimedium Refractive Stereo Calibration,” IEEE Transactions on Industrial Electronics 71, no. 7 (2024): 7452–7462, 10.1109/TIE.2023.3269875.

[32] J. Di , Z. Dugonjic , W. Fu , et al., “Using Fiber Optic Bundles to Miniaturize Vision‐Based Tactile Sensors,” IEEE Transactions on Robotics 41 (2025): 62–81, 10.1109/TRO.2024.3492375.

[33] Z. Zhao , W. Li , Y. Li , et al., “Embedding High‐Resolution Touch Across Robotic Hands Enables Adaptive Human‐Like Grasping,” Nature Machine Intelligence 7, no. 6 (2025): 889–900, 10.1038/s42256-025-01053-3.

[34] C. L. de Korte , H. H. G. Hansen , and A. F. W. van der Steen , “Vascular Ultrasound for Atherosclerosis Imaging,” Interface Focus 1, no. 4 (2011): 565–575. 22866231 10.1098/rsfs.2011.0024 PMC3262270

[35] D. F. Gomes , P. Paoletti , and S. Luo , “Generation of GelSight Tactile Images for Sim2Real Learning,” IEEE Robotics and Automation Letters 6, no. 2 (2021): 4177–4184, 10.1109/LRA.2021.3063925.

[36] Y. Zhao , K. Qian , B. Duan , and S. Luo , “FOTS: A Fast Optical Tactile Simulator for Sim2Real Learning of Tactile‐Motor Robot Manipulation Skills,” IEEE Robotics and Automation Letters 9, no. 6 (2024): 5647–5654, 10.1109/LRA.2024.3396665.

[37] A. Dosovitskiy , L. Beyer , A. Kolesnikov , et al., “An Image is Worth 16x16 words: Transformers for Image Recognition at Scale,”*arXiv* (2021): arXiv:2010.11929, http://arxiv.org/abs/2010.11929.

[38] J. Kim , M. Lee , H. J. Shim , et al., “Stretchable Silicon Nanoribbon Electronics for Skin Prosthesis,” Nature Communications 5, no. 1 (2014): 5747, 10.1038/ncomms6747. 25490072

[39] G. Li , S. Liu , L. Wang , and R. Zhu , “Skin‐Inspired Quadruple Tactile Sensors Integrated on a Robot Hand Enable Object Recognition,” Science Robotics 5, no. 49 (2020): eabc8134, 10.1126/scirobotics.abc8134. 33328298

[40] S. Dong , T. Yang , Y. Lou , et al., “A High‐Precision Miniature 3‐D Tactile Force Sensor Based on Fiber Bragg Grating for Minimally Invasive Surgery,” IEEE Transactions on Instrumentation and Measurement 74 (2025): 1–10.42146727

[41] M. Mir , J. Chen , A. Patel , et al., “A Minimally Invasive Robotic Tissue Palpation Device,” IEEE Transactions on Biomedical Engineering 71, no. 6 (2024): 1958–1968, 10.1109/TBME.2023.3344335. 38261510 PMC11178256

[42] T. Liu , X. Zhang , C. Zhang , et al., “Robotic Intracorporeal Palpation with a Miniature Force‐Sensing Probe for Minimally Invasive Surgery,” IEEE Transactions on Instrumentation and Measurement 74 (2025): 1–10.42146727

[43] F. Campisano , S. Ozel , A. Ramakrishnan , et al., “Towards a Soft Robotic Skin for Autonomous Tissue Palpation,” in 2017 IEEE International Conference on Robotics and Automation (ICRA) (IEEE, 2017), 6150–6155.

[44] S. Luo , N. F. Lepora , W. Yuan , K. Althoefer , G. Cheng , and R. Dahiya , “Tactile Robotics: An Outlook,” IEEE Transactions on Robotics 41 (2025): 5564–5583, 10.1109/TRO.2025.3608686.

[45] B. Winstone , G. Griffiths , C. Melhuish , T. Pipe , and J. Rossiter , “TACTIP – Tactile Fingertip Device, Challenges in Reduction of Size to Ready for Robot Hand Integration,” in 2012 IEEE International Conference on Robotics and Biomimetics (ROBIO) (IEEE, 2012), 160–166.

[46] D. F. Gomes , Z. Lin , and S. Luo , “GelTip: A Finger‐Shaped Optical Tactile Sensor for Robotic Manipulation,” in 2020 IEEE/RSJ International Conference on Intelligent Robots and Systems (IROS) (IEEE, 2020), 9903–9909.

[47] P. Rayamane , Z. Ji , and M. Packianather , “Design and Development of a Robust Vision‐Based Tactile Sensor,” in 2022 IEEE/ASME International Conference on Advanced Intelligent Mechatronics (AIM) (IEEE, 2022), 1417–1423, https://ieeexplore.ieee.org/document/9863285.

[48] C. Lin , Z. Lin , S. Wang , and H. Xu , “DTact: A Vision‐Based Tactile Sensor That Measures High‐Resolution 3D Geometry Directly from Darkness,” in 2023 IEEE International Conference on Robotics and Automation (ICRA) (IEEE, 2023), 10359–10366.

[49] C. Lin , H. Zhang , J. Xu , L. Wu , and H. Xu , “9DTact: A Compact Vision‐Based Tactile Sensor for Accurate 3D Shape Reconstruction and Generalizable 6D Force Estimation,” IEEE Robotics and Automation Letters 9, no. 2 (2024): 923–930, 10.1109/LRA.2023.3339397.

[50] C. Lu , Z. Liang , D. Stoyanov , and A. Stilli , “GelPoLight: A Novel Visual‐Tactile Sensor Based on Photometric Stereo with Point Lighting,” IEEE Sensors Journal 25, no. 6 (2025): 9575–9584, 10.1109/JSEN.2025.3535080.

[51] M. A. Mirzaee , H.‐J. Huang , and W. Yuan , “GelBelt: A Vision‐Based Tactile Sensor for Continuous Sensing of Large Surfaces,” IEEE Robotics and Automation Letters 10, no. 2 (2025): 2016–2023, 10.1109/LRA.2025.3527306.

[52] J. Xu , L. Wu , C. Lin , D. Zhao , and H. Xu , “DTactive: A Vision‐Based Tactile Sensor with Active Surface,” in 2025 IEEE/RSJ International Conference on Intelligent Robots and Systems (IROS) (IEEE, 2025), 21664–21670, https://ieeexplore.ieee.org/document/11247152.

[53] J. Jiang , X. Zhang , D. F. Gomes , T.‐T. Do , and S. Luo , “RoTipBot: Robotic Handling of Thin and Flexible Objects Using Rotatable Tactile Sensors,” IEEE Transactions on Robotics 41 (2025): 3684–3702, 10.1109/TRO.2025.3576951.

[54] Z. Wu , Y. Lin , Y. Zhao , et al., “ViTacGen: Robotic Pushing with Vision‐to‐Touch Generation,” IEEE Robotics and Automation Letters 10, no. 11 (IEEE, 2025): 12269–12276, 10.1109/LRA.2025.3621941.

[55] Y. Zhao , X. Jing , K. Qian , D. F. Gomes , and S. Luo , “Skill Generalization of Tubular Object Manipulation with Tactile Sensing and Sim2Real Learning,” Robotics and Autonomous Systems 160 (2023): 104321, 10.1016/j.robot.2022.104321.

[56] J. Ohayon , G. Finet , S. Le Floc'h , et al., “Biomechanics of Atherosclerotic Coronary Plaque: Site, Stability and In Vivo Elasticity Modeling,” Annals of Biomedical Engineering 42, no. 2 (2014): 269–279, 10.1007/s10439-013-0888-1. 24043605 PMC4722860

[57] B. R. Kwak , M. Bäck , M.‐L. Bochaton‐Piallat , et al., “Biomechanical Factors in Atherosclerosis: Mechanisms and Clinical Implications,” European Heart Journal 35, no. 43 (2014): 3013–3020, 10.1093/eurheartj/ehu353. 25230814 PMC4810806

[58] S. Z. Gu and M. R. Bennett , “Plaque Structural Stress: Detection, Determinants and Role in Atherosclerotic Plaque Rupture and Progression,” Frontiers in Cardiovascular Medicine 9 (2022): 875413, 10.3389/fcvm.2022.875413. 35872913 PMC9300846

[59] A. Tziotziou , E. Hartman , S.‐A. Korteland , et al., “Mechanical Wall Stress and Wall Shear Stress Are Associated with Atherosclerosis Development in Non‐Calcified Coronary Segments,” Atherosclerosis 387 (2023): 117387, 10.1016/j.atherosclerosis.2023.117387. 38029610

[60] S. Dong , Z. Liu , Y. Lou , et al., “A High‐Precision Miniature Triaxial FBG Force Sensor for Detecting Tissue Anomalies,” Journal of Lightwave Technology 42, no. 17 (2024): 6143–6152, 10.1109/JLT.2024.3403206.

[61] T. Li , C. Shi , and H. Ren , “A High‐Sensitivity Tactile Sensor Array Based on Fiber Bragg Grating Sensing for Tissue Palpation in Minimally Invasive Surgery,” IEEE/ASME Transactions on Mechatronics 23, no. 5 (2018): 2306–2315, 10.1109/TMECH.2018.2856897.

[62] W. Lai , L. Cao , R. X. Tan , et al., “Force Sensing with 1 mm Fiber Bragg Gratings for Flexible Endoscopic Surgical Robots,” IEEE/ASME Transactions on Mechatronics 25, no. 1 (2020): 371–382, 10.1109/TMECH.2019.2951540.

[63] C. Hou , K. Wang , F. Wang , et al., “A Highly Integrated 3D MEMS Force Sensing Module with Variable Sensitivity for Robotic‐Assisted Minimally Invasive Surgery,” Advanced Functional Materials 33, no. 43 (2023): 2302812, 10.1002/adfm.202302812.

[64] U. Kim , Y. B. Kim , J. So , D.‐Y. Seok , and H. R. Choi , “Sensorized Surgical Forceps for Robotic‐Assisted Minimally Invasive Surgery,” IEEE Transactions on Industrial Electronics 65, no. 12 (2018): 9604–9613, 10.1109/TIE.2018.2821626.

[65] X. Huo , B. Liu , and Z. Wu , “Recent Advances of Flexible Pressure Tactile Sensors: Sensing Mechanisms, Performance Breakthroughs, and Intelligent Applications,” Advanced Materials Technologies 11, no. 5 (2026): e01837, 10.1002/admt.202501837.

[66] S. Wang , Z. Song , L. Chen , X. Wang , and L. Xie , “High‐Performance 3‐D Liquid‐Metal Tactile Sensing System with Data‐Driven Signal Decoding for Dynamic Force Estimation,” IEEE Transactions on Instrumentation and Measurement 75 (2026): 1–10, 10.1109/TIM.2026.3670567.

[67] M. K. Johnson and E. H. Adelson , “Retrographic Sensing for the Measurement of Surface Texture and Shape,” in 2009 IEEE Conference on Computer Vision and Pattern Recognition (IEEE, 2009), 1070–1077.

[68] B. Ward‐Cherrier , N. Pestell , L. Cramphorn , et al., “The TacTip Family: Soft Optical Tactile Sensors with 3D‐Printed Biomimetic Morphologies,” Soft Robotics 5, no. 2 (2018): 216–227, 10.1089/soro.2017.0052. 29297773 PMC5905869

[69] X. Zhang , J. Jiang , and S. Luo , “RoTip: A Finger‐Shaped Tactile Sensor with Active Rotation,” arXiv (2024): arXiv:2410.01085.

[70] X. Zhang , T. Yang , D. Zhang , and N. F. Lepora , “TacPalm: A Soft Gripper with a Biomimetic Optical Tactile Palm for Stable Precise Grasping,” IEEE Sensors Journal 24, no. 22 (2024): 38402–38416.

[71] W. Fan , H. Li , W. Si , S. Luo , N. Lepora , and D. Zhang , “ViTacTip: Design and Verification of a Novel Biomimetic Physical Vision‐Tactile Fusion Sensor,” in 2024 IEEE International Conference on Robotics and Automation (ICRA) (IEEE, 2024), 1056–1062.

[72] E. Donlon , S. Dong , M. Liu , J. Li , E. Adelson , and A. Rodriguez , “GelSlim: A High‐Resolution, Compact, Robust, and Calibrated Tactile‐Sensing Finger,” in 2018 IEEE/RSJ International Conference on Intelligent Robots and Systems (IROS) (IEEE, 2018), 1927–1934.

[73] D. Ma , E. Donlon , S. Dong , and A. Rodriguez , “Dense Tactile Force Distribution Estimation Using GelSlim and Inverse FEM,” arXiv (2019): arXiv:1904.02862.

[74] Y. Gao , S. Zhang , W. Wan , B. Fang , F. Sun , and K. Harada , “Development of a Miniature Photometric Vision‐Based Tactile Sensor,” IEEE Sensors Journal 24, no. 20 (2024): 32053–32064.

[75] J. Zhao , N. Kuppuswamy , S. Feng , B. Burchfiel , and E. Adelson , “PolyTouch: A Robust Multi‐Modal Tactile Sensor for Contact‐rich Manipulation Using Tactile‐Diffusion Policies,” arXiv (2025): arXiv:2504.12345.

