# Supplementary material for: EndoTac: An Endoscopic Camera‐Based Tactile Sensor with High Sensitivity for Minimally Invasive Surgery
Source: Adv Sci (Weinh). 2026 Jul 31:e76921. Online ahead of print. doi: 10.1002/advs.76921 (PMC13427239; doi:10.1002/advs.76921)
Supplement: Supplementary file 1 — Supporting File 1: advs76921‐sup‐0001‐SuppMat.pdf. [file ADVS-9999-e76921-s002.pdf]

1 Appendix

1.1 Mirror Geometry Analysis

This appendix describes the geometric analysis used to guide the mirror design of EndoTac. In the two-dimensional side-view model, the mirror is described by three parameters: the terminal mirror angle α, the radius of curvature of the cylindrical mirror section r, and the length of the flat mirror section d. These parameters define the reflective profile between the endoscopic camera and the side-facing sensing membrane. The camera position, camera field of view, sensing membrane position, and probe housing dimensions are treated as fixed design constraints. Therefore, the mirror geometry can be represented as

$$\mathbf{x} = [\alpha, r, d]. \tag{1}$$

As illustrated in Figure S1, α defines the terminal angle of the mirror, r defines the radius of curvature of the cylindrical section, and d defines the flat-section length. These three parameters determine how the camera field of view is reflected onto the sensing membrane.

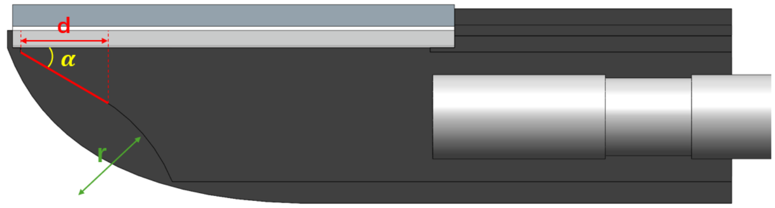

Figure S1: Geometric parameters used to describe the mirror profile in the side-view model. The mirror geometry is defined by the terminal mirror angle α, the radius of curvature of the cylindrical mirror section r, and the flat-section length d.

The terminal mirror angle α is first considered because the mirror tip has the smallest local angle relative to the sensing surface. This angle affects the boundary of the reflected camera field of view and therefore influences the observable sensing length on the membrane. To describe this effect, the terminal part of the mirror is treated locally as a flat reflective surface. For a camera field of view of 60° and a channel height d_c, the projected observable sensing length can be approximated as

$$L(\alpha) = \frac{d_c}{\tan(\alpha)} + \frac{d_c}{\tan(150^\circ - 2\alpha)}. \tag{2}$$

This expression shows that, in the considered design range, reducing α increases the projected observable sensing length. However, α is also constrained by the viewing quality at the boundary of the camera field of view. If the reflected boundary ray reaches the membrane at a shallow angle, the corresponding region is viewed in a grazing configuration, which causes foreshortening and reduces the effective spatial sampling of the tactile surface.

Optical metrology studies commonly define the incidence angle as the angle between the viewing ray and the surface normal. Larger incidence angles have been shown to reduce signal quality, increase footprint elongation, and degrade measurement precision [1]. In a single-shot laser scanning study, Pexman and Robson recommended constraining the incidence angle to less than 30° from the surface normal for an interior aerospace surface [2]. Since the viewing angle γ used here is measured from the sensing surface rather than from the surface normal, this corresponds to

$$\gamma_{\text{min}} = 90^\circ - 30^\circ = 60^\circ. \tag{3}$$

Therefore, the boundary viewing angle was constrained by

$$\gamma_{\text{boundary}} \geq 60^\circ. \tag{4}$$

For the local flat-tip approximation, the boundary viewing angle is related to the terminal mirror angle by

$$\gamma_{\text{boundary}} = 2\alpha. \tag{5}$$

Applying the viewing-angle criterion gives

$$2\alpha \geq 60^\circ, \tag{6}$$

and therefore

$$\alpha \geq 30°. \quad (7)$$

Since a smaller α gives a larger projected sensing length according to Eq. 2, the terminal mirror angle was selected as the smallest value satisfying this viewing-angle constraint:

$$\alpha = 30°. \quad (8)$$

After fixing $\alpha = 30°$, the remaining geometry of the curved mirror section is constrained by the target observable sensing length. In the current design, the target sensing length is

$$L_0 = 16 \text{ mm}. \quad (9)$$

With the channel height

$$d_c = 6 \text{ mm}, \quad (10)$$

this can also be written as

$$L_0 = \frac{8}{3} d_c. \quad (11)$$

The boundary of the mirror should be able to reflect the edge of this 16 mm sensing region back into the camera field of view. To express this condition, the endpoint of the curved mirror is described using two auxiliary parameters, as shown in Figure S2, the horizontal distance m from the curved endpoint to the sensing area boundary, and the local endpoint angle β.

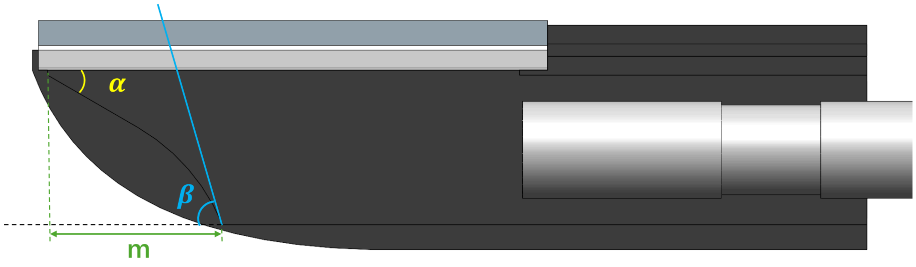

Figure S2: Geometric construction used to define the curved-mirror endpoint. The endpoint is described by the distance m and the local mirror angle β, after fixing the terminal mirror angle as $\alpha = 30°$.

Let the horizontal distance between the camera-side reference point and the curved-mirror endpoint be

$$x = L_0 - m = 16 - m. \quad (12)$$

From the reflection geometry, the two relevant ray angles are

$$\theta_1 = \tan^{-1}\left(\frac{6}{16 - m}\right), \quad (13)$$

and

$$\theta_2 = \tan^{-1}\left(\frac{3}{16 - m}\right). \quad (14)$$

The endpoint angle β is then obtained from the local reflection condition as

$$\beta(m) = 90° - \frac{1}{2}\left[\tan^{-1}\left(\frac{6}{16 - m}\right) + \tan^{-1}\left(\frac{3}{16 - m}\right)\right]. \quad (15)$$

This equation gives the required endpoint angle β for a given endpoint location m. As m increases, β decreases. The feasible range of m is constrained by the geometry of the 30° terminal slope and by the physical constrains of the curved mirror. The terminal-slope geometry gives

$$m < \frac{6}{\tan 30°} = 10.39 \text{ mm}. \quad (16)$$

A further bound is introduced because, when β becomes too large, the circular curved section extends outside the feasible mirror boundary defined by the 30° terminal slope. From the geometric construction, this limiting condition occurs at approximately

$$\beta_{\max} \approx 67.6^\circ, \tag{17}$$

which corresponds through Eq. 15 to

$$m \approx 5.25 \text{ mm}. \tag{18}$$

The feasible interval for the curved endpoint is therefore

$$5.25 \text{ mm} \leq m < 10.39 \text{ mm}, \tag{19}$$

with the corresponding endpoint-angle range

$$52.3^\circ < \beta \leq 67.6^\circ. \tag{20}$$

Within this feasible interval, the final mirror geometry was selected by considering the angular to spatial mapping between the camera field of view and the sensing membrane. A ray emitted from the camera origin with angle θ can be written as

$$y = \tan(\theta)x, \tag{21}$$

where

$$-30^\circ \leq \theta \leq 30^\circ. \tag{22}$$

For each candidate value of m, the corresponding β is calculated from Eq. 15. The mirror profile is then defined by the cylindrical curved section and the terminal flat section. The reflected ray is calculated using the local tangent angle at the mirror-intersection point, and the position where this reflected ray reaches the sensing surface at $y = 3$ is denoted as $x(\theta; m)$.

To quantify the nonuniformity of the reflected mapping, the local angular to spatial mapping scale is defined as

$$q(\theta; m) = \left| \frac{dx(\theta; m)}{d\theta} \right|. \tag{23}$$

This quantity represents the physical sensing length covered by one unit angle of the camera field of view. A larger value indicates that a fixed angular interval corresponds to a longer region on the sensing membrane, which implies lower effective spatial sampling. Therefore, for each candidate m, the peak mapping scale was evaluated as

$$q_{\max}(m) = \max_{\theta} \left| \frac{dx(\theta; m)}{d\theta} \right|, \qquad -30^\circ \leq \theta \leq 30^\circ. \tag{24}$$

The endpoint position was selected by minimizing this peak mapping scale within the feasible interval:

$$m = \arg\min_{m} q_{\max}(m), \qquad 5.25 \text{ mm} \leq m < 10.39 \text{ mm}. \tag{25}$$

A numerical search was then used to evaluate $q_{\max}(m)$ over the feasible range. As shown in Figure S3, the minimum occurs at approximately

$$m \approx 8.8 \text{ mm}. \tag{26}$$

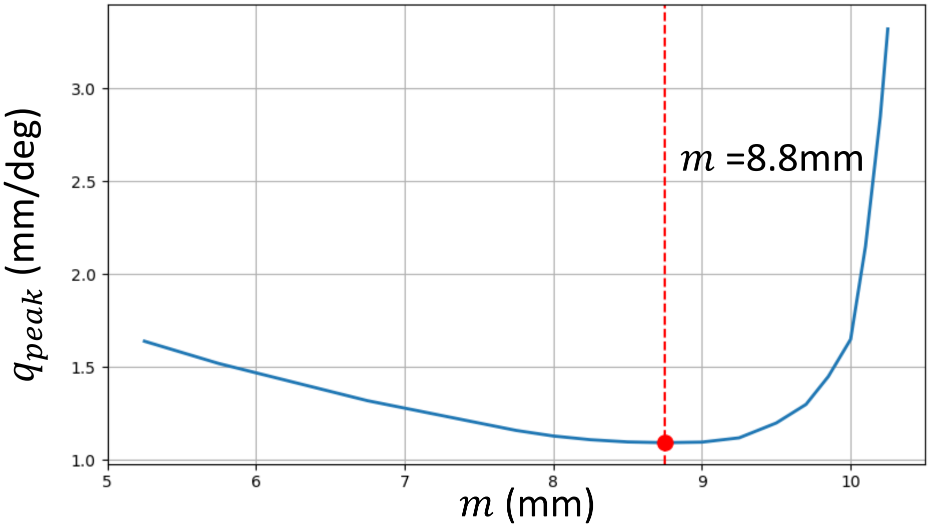

Figure S3: Peak angular to spatial mapping scale $q_{\max}(m)$ over the feasible range of m. The selected endpoint position is approximately $m = 8.8$ mm.

Substituting this value into Eq. 15 gives

$$\beta \approx 58.8^\circ. \tag{27}$$

At this geometry, the peak angular to spatial mapping scale is approximately

$$q_{\max} \approx 1.09 \text{ mm/degree}. \tag{28}$$

The auxiliary endpoint parameters m and β are then converted back to the original mirror-geometry parameterization. The corresponding radius of curvature is

$$r \approx 6.44 \text{ mm}, \tag{29}$$

and the corresponding flat-section length is

$$d \approx 6.51 \text{ mm}. \tag{30}$$

Thus, under the defined geometric constraints and angular to spatial mapping criterion, the selected mirror geometry is

$$\mathbf{x} = [\alpha, r, d] = [30^\circ, \text{ 6.44 mm, 6.51 mm}]. \tag{31}$$

Here, α is the terminal mirror angle, r is the radius of curvature of the cylindrical mirror section, and d is the flat-section length. The auxiliary parameters m and β are used only to define the endpoint of the curved section during the geometric construction.

1.2 Baseline Model Comparison for Vessel Deformation Estimation

To further evaluate the selection of ResNet-18 as the deformation-estimation model, two additional baseline models were implemented and compared with the ResNet-18 regressor: a convolutional neural network (CNN) and a lightweight Vision Transformer (ViT). The purpose of this comparison was to assess whether ResNet-18 provided improved prediction accuracy and generalization compared with a compact convolutional model and an attention-based visual model.

The CNN baseline [3] was designed as a lightweight convolutional regressor. It consisted of five convolutional blocks followed by global average pooling and a two-layer multilayer perceptron (MLP) regression head. A sigmoid output layer was used to predict the normalized vessel deformation. This model was included to evaluate whether a smaller task-specific CNN could achieve comparable performance to the ResNet-18 model.

The lightweight ViT baseline [4] was implemented as an attention-based regressor. The input tactile image was divided into 16×16 patches, which were projected into a 192-dimensional embedding space. The model used six transformer encoder layers, three attention heads, a class token, positional embedding, and an MLP regression head with a sigmoid output. This baseline was included to compare ResNet-18 with a non-convolutional architecture for the same vessel deformation regression task.

The three models were evaluated on the seen test set and two independent unseen test sets. The unseen 8-mm vessel test set was collected from a vessel object with 8-mm vessel diameter. The unseen 6-mm molded vessel test set was collected

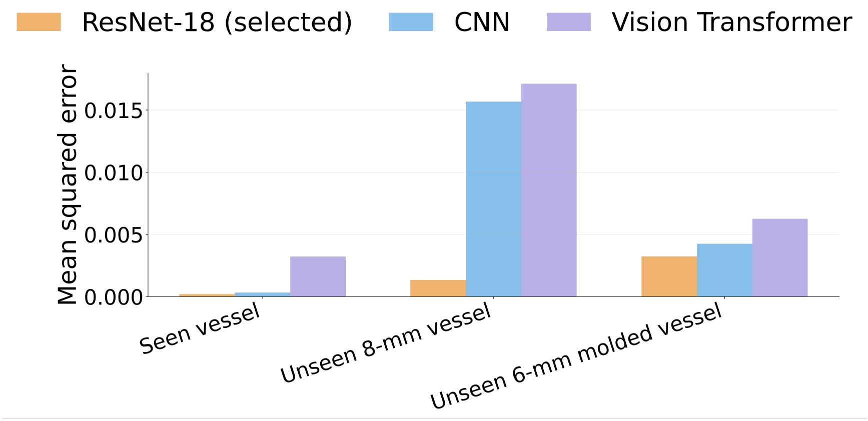

Figure S4: Accuracy comparison between ResNet-18 and baseline models for vessel deformation estimation. The comparison of MSE across the seen test set, and unseen test sets. ResNet-18 achieved the lowest error and showed better generalization to unseen vessel conditions.

from a molded vessel object that was not included in training. These unseen-vessel test sets were used to assess model generalization to vessel conditions outside the training data.
As shown in Figure S4, ResNet-18 achieved the lowest error on the seen test set. It also showed stronger generalization on the two unseen-vessel test sets. On the unseen 8-mm vessel test set, both the CNN and ViT baselines showed a clear increase in prediction error, whereas the ResNet-18 model maintained lower MSE. On the unseen 6-mm molded vessel test set, ResNet-18 also achieved the lowest error among the three models.
These results indicate that ResNet-18 provided the best overall deformation-estimation performance among the evaluated architectures.

References

[1] S. Soudarissanane, R. Lindenbergh, M. Menenti, P. Teunissen, *ISPRS Journal of Photogrammetry and Remote Sensing* **2011**, *66*, 4 389.

[2] K. Pexman, S. Robson, In *The International Archives of the Photogrammetry, Remote Sensing and Spatial Information Sciences*, volume XLVIII-2/W7-2024. **2024** 113–120.

[3] W. Yuan, S. Dong, E. H. Adelson, *Sensors* **2017**, *17*, 12 2762.

[4] A. Dosovitskiy, L. Beyer, A. Kolesnikov, D. Weissenborn, X. Zhai, T. Unterthiner, M. Dehghani, M. Minderer, G. Heigold, S. Gelly, J. Uszkoreit, N. Houlsby, An image is worth 16x16 words: Transformers for image recognition at scale, **2021**, URL `http://arxiv.org/abs/2010.11929`, ArXiv:2010.11929 [cs.CV].
